# Polygenic Risk Score Modifies Prostate Cancer Risk of Pathogenic Variants in Men of African Ancestry

**DOI:** 10.1158/2767-9764.CRC-23-0022

**Published:** 2023-12-14

**Authors:** Raymond W. Hughley, Marco Matejcic, Ziwei Song, Xin Sheng, Peggy Wan, Lucy Xia, Steven N. Hart, Chunling Hu, Siddhartha Yadav, Alexander Lubmawa, Vicky Kiddu, Frank Asiimwe, Colline Amanya, George Mutema, Kuteesa Job, Mbaaga K. Ssebakumba, Sue A. Ingles, Ann S. Hamilton, Fergus J. Couch, Stephen Watya, David V. Conti, Burcu F. Darst, Christopher A. Haiman

**Affiliations:** 1 Center for Genetic Epidemiology, Department of Population and Public Health Sciences, Keck School of Medicine, University of Southern California, Los Angeles, California.; 2 Department of Population and Public Health Sciences, Keck School of Medicine, University of Southern California, Los Angeles, California.; 3 Department of Quantitative Health Sciences, Mayo Clinic, Rochester, Minnesota.; 4 Department of Laboratory Medicine and Pathology, Mayo Clinic, Rochester, Minnesota.; 5 Department of Oncology, Mayo Clinic, Rochester, Minnesota.; 6 Uro Care, Kampala, Uganda.; 7 Mulago Hospital, Kampala, Uganda.; 8 Makerere University College of Health Sciences, Kampala, Uganda.; 9 SurgPath, Kampala, Uganda.; 10 Kagando Hospital, Kasese, Uganda.; 11 Public Health Sciences, Fred Hutchinson Cancer Center, Seattle, Washington.

## Abstract

Prostate cancer risk is influenced by rare and common germline variants. We examined the aggregate association of rare germline pathogenic/likely pathogenic/deleterious (P/LP/D) variants in *ATM, BRCA2, PALB2,* and *NBN* with a polygenic risk score (PRS) on prostate cancer risk among 1,796 prostate cancer cases (222 metastatic) and 1,424 controls of African ancestry. Relative to P/LP/D non-carriers at average genetic risk (33%–66% of PRS), men with low (0%–33%) and high (66%–100%) PRS had Odds Ratios (ORs) for overall prostate cancer of 2.08 [95% confidence interval (CI) = 0.58–7.49] and 18.06 (95% CI = 4.24–76.84) among P/LP/D carriers and 0.57 (95% CI = 0.46–0.71) and 3.02 (95% CI = 2.53–3.60) among non-carriers, respectively. The OR for metastatic prostate cancer was 2.73 (95% CI = 0.24–30.54) and 28.99 (95% CI = 4.39–191.43) among P/LP/D carriers and 0.54 (95% CI = 0.31–0.95) and 3.22 (95% CI = 2.20–4.73) among non-carriers, for men with low and high PRS, respectively. Lifetime absolute risks of overall prostate cancer increased with PRS (low to high) from 9.8% to 51.5% in P/LP/D carriers and 5.5% to 23.9% in non-carriers. Lifetime absolute risks of metastatic prostate cancer increased with PRS from 1.9% to 18.1% in P/LP/D carriers and 0.3% to 2.2% in non-carriers These findings suggest that assessment of prostate cancer risk for rare variant carriers should include PRS status.

**Significance:**

These findings highlight the importance of considering rare and common variants to comprehensively assess prostate cancer risk in men of African ancestry.

## Introduction

Pathogenic variants in DNA repair genes are associated with risk of advanced prostate cancer and determine eligibility for targeted therapies (1–5). We recently reported in European ancestry men that prostate cancer risk associated with carrying pathogenic variants in established prostate cancer risk genes is more accurately determined when incorporating information regarding a man's genetic risk profile based on common risk variants, as measured by a polygenic risk score (PRS; ref. 6). Pathogenic variants and a prostate cancer PRS have been separately shown to impact prostate cancer risk in African ancestry men (5, 7–10); however, the combined association of exceedingly rare pathogenic variants and common variants found through genome-wide association study has not been explored in men of African ancestry. Understanding the degree to which PRS modifies risk for African ancestry rare variant carriers may inform risk assessment and have clinical value. We investigated the combined association of rare variants in *BRCA2, ATM, NBN,* and *PALB2* (5) and common variants, evaluated with a multi-ancestry prostate cancer PRS (7), on prostate cancer risk in a study of 3,220 men of African ancestry.

## Materials and Methods

### Participants

This study included 1,796 prostate cancer cases (903 aggressive, and 735 nonaggressive) and 1,424 controls of African ancestry from the Multiethnic Cohort (MEC; 735 cases, 965 controls; ref. 11), the Los Angeles Study of Aggressive prostate cancer (LAAPC; 77 cases; ref. 12), cases recruited in Los Angeles County (LAC; 475 cases; ref. 5), and the Uganda Prostate Cancer Study (UGPCS; 510 cases and 459 controls; ref. 13) with genome-wide genotyping (7) and targeted gene panel sequencing (ref. 5; Table 1). Men with a higher Gleason score and stage were overselected from these studies to increase power to identify rare variants and genes associated with more aggressive disease. Incident prostate cancer cases and information on histologic status of disease for the MEC, LAC, and LAAPC were identified through linkage to Surveillance, Epidemiology, and End Results (SEER) Program cancer registries in California. In UGPCS, incident prostate cancer cases were recruited from 13 hospitals/clinics across the country, and 487 controls were enrolled from nonurological clinics (e.g., surgery). All UGPCS controls had a PSA level <4 ng/mL. Participants all signed a written consent, and the study protocol was approved by the Institutional Review Boards of the University of Southern California (Los Angeles, CA) and Makerere University (Kampala, Uganda). The human investigations were performed after approval by a local Human Investigations Committee and in accordance with an assurance filed with and approved by the Department of Health and Human Services, where appropriate.

**TABLE 1:** Descriptive characteristics of prostate cancer cases and controls

	African American		Uganda	
Variable	Controls *N* = 965	Cases *N* = 1,286	Controls *N* = 459	Cases *N* = 510
Median age (IQR), years	71 (67–76)	67 (62–73)	65 (60–70)	70 (65–78)
Age, years				
≤65	149 (15.4%)	517 (40.2%)	261 (56.9%)	140 (27.5%)
>65	816 (84.6%)	769 (59.8%)	198 (43.1%)	370 (72.5%)
First-degree family history of PCa				
No	771 (79%)	925 (71.9%)	419 (91.3%)	324 (63.5%)
Yes	97 (10%)	262 (20.3%)	10 (2.2%)	46 (9%)
Gleason score^a^				
≤7		942 (73.3%)		168 (32.9%)
8–10		326 (25.3%)		154 (30.2%)
Stage^a^				
Localized		935 (72.7%)		
Metastatic		70 (5.4%)		
Regional		235 (18.3%)		
PSA level, ng/mL^a^				
<50				103 (20.2%)
50–100				84 (16.5%)
>100				152 (29.8%)
Aggressiveness^a^^,^^b^				
Aggressive		591 (46%)		312 (61.2%)
Nonaggressive		695 (54%)		40 (7.8%)

NOTE: Aggressive disease was defined as Gleason score >7 or nonlocalized disease (regional or metastatic) or PSA level >50 ng/mL or death from prostate cancer.

Abbreviations: IQR, interquartile range; PCa, prostate cancer; PSA, prostate-specific antigen.

^a^Numbers do not sum to the total sample size due to missing data.

^b^Nonaggressive disease was defined as Gleason score ≤7 and localized disease (for African Americans) or PSA level ≤50 ng/mL (for Ugandans).

Aggressive disease was defined as cases with nonlocalized disease, Gleason >7 tumors, or those who died because of prostate cancer (Gleason score >7 or PSA level >50 ng/mL was used for UGPCS, as staging information was not available; ref. 14). Less aggressive disease (labeled “nonaggressive”) was defined as cases with localized disease and Gleason ≤7 tumors (Gleason score ≤7 and PSA ≤50 ng/mL for UGPCS). UGPCS cases with a PSA level >100 were classified as having metastatic disease (15–17).

### Targeted Gene Sequencing and Genome-wide Genotyping

Gene sequencing and quality control procedures have been described previously (5). Briefly, next-generation sequencing was performed using an Illumina HiSeq 4000 with 150 bp paired end reads. Genomic DNA libraries were prepared using a custom QIAseq amplicon-based targeted panel of all coding regions and essential splice sites. Reads were aligned to the human reference genome (GRCh37) using BWA-MEM v0.7.10. The Genome Atlas Toolkit (GATK v3.4-46) was utilized for sequence realignment and recalibration, joint sample calling, and evaluation of depth of coverage.

Genome-wide genotyping was performed using the Illumina Human 1M and Illumina OncoArray in the MEC, LAC, and LAAPC studies and the Illumina OncoArray in UGPCS. All studies were imputed to phase III of the 1000 Genomes Project reference panel (18). Genotyping and postimputation quality control details are summarized elsewhere (7).

### Criteria for Variant Pathogenicity

Analyses focused on rare (minor allele frequency <1%) germline pathogenic, likely pathogenic, or deleterious (P/LP/D) variants (*n* = 51) in *BRCA2, ATM, NBN,* and *PALB2* (Supplementary Table S1). As described previously (5), P/LP/D variants were defined as variants predicted to result in protein truncation or to significantly alter the protein sequence (frameshift insertion/deletion, gain of stop codon, or loss of essential splice site donor/acceptor) and missense variants (nonsynonymous codon change and exon start/end codon change) that were reported as pathogenic or likely pathogenic in ClinVar (19) by one or more clinical laboratories (Ambry, SCRP, InVitae, GeneDX, Emory, and InSiGHT). Missense variants not identified as pathogenic or likely pathogenic in ClinVar were annotated with dbNSFP version 3.3a (20) using five *in silico* algorithms (Polyphen2-HumDiv, PolyPhen2-HumVar, LRT, Mutation Taster, and SIFT) to predict potential functional effects (21) and determine P/LP/D status. Variants in introns, 5′-UTR (untranslated region), 3′-UTR, in-frame insertions/deletions, synonymous variants, and those located 2 bp outside of the consensus splice site according to CAVA (22) were excluded. *BRCA2*, *ATM*, *NBN*, and *PALB2* P/LP/D variants were found in 1% of nonaggressive cases, 3.3% of aggressive cases, 4.2% of metastatic cases, and 0.6% of controls.

### PRS

The prostate cancer PRS was calculated on the basis of a previously developed multi-ancestry PRS of 269 risk variants (7). Of the 269 variants, 254 were polymorphic in the current dataset (minor allele frequency >1% in controls). The PRS was calculated as a weighted sum of the number of risk alleles carried by each participant, using the previously reported variant-specific multi-ancestry weights (7), and analyzed as high- (66.7%–100%), intermediate- (33.3%–66.7%), and low- (0%–33.3%) risk groups (PRS tertiles) based on the PRS distribution among controls.

### Statistical Analysis

The combined effect of the PRS and carrier status on prostate cancer risk was evaluated by aggregating across P/LP/D variants in *BRCA2*, *ATM*, *NBN*, and *PALB2*. Men were classified on the basis of the combination of carrier status and PRS in the following six categories: low PRS non-carriers, low PRS carriers, intermediate PRS non-carriers, intermediate PRS carriers, high PRS non-carriers, and high PRS carriers. Logistic regression models were used to calculate ORs for prostate cancer for each of these categories, adjusting for age, study, and the first 10 principal components of ancestry to account for potential population stratification, using the intermediate PRS non-carrier category as the reference group. Outcomes evaluated included overall prostate cancer, metastatic, aggressive, and nonaggressive disease versus controls.

### Absolute Risk Estimation

Absolute risks of prostate cancer were estimated (7) for a given age for each PRS category, carrier status, and the combined PRS category and carrier status variable using the ORs from each respective analysis (as described above), combined with mortality and incidence rates for Black men in the United States (23, 24), while accounting for competing causes of death. Because our study population was enriched for aggressive cases, for overall prostate cancer absolute risk estimates, we calculated ORs that weighted aggressive and nonaggressive prostate cancer ORs based on the expected proportions of aggressive and nonaggressive prostate cancer cases in a general Black population (37% and 63%, respectively, based on data from the African Ancestry Prostate Cancer Consortium; Supplementary Tables S2 and S3). For metastatic prostate cancer absolute risk, we used metastatic prostate cancer ORs (Supplementary Tables S4–S6). Overall and metastatic prostate cancer incidence rates were obtained from the SEER Program (23, 24). Population frequencies for combined PRS P/LP/D groups were represented by weighted average frequencies calculated from the frequency of cases and controls in each PRS P/LP/D group within the study weighted by the frequency of prostate cancer in African American men (16.7%; ref. 25) for combined PRS P/LP/D groups. Absolute risk confidence intervals (CI) were generated using estimates averaged from 1,000 iterations of the Monte Carlo sampling for a specific age for PRS categories and carrier status.

### Data Availability Statement

The genotype data and relevant covariate information (e.g., ancestry, country, and principal components) used in this study are deposited in dbGaP under accession codes phs000838.v1.p1, phs000306.v4.p1, and phs001391.v1.p1.

## Results

**FIGURE 1:**
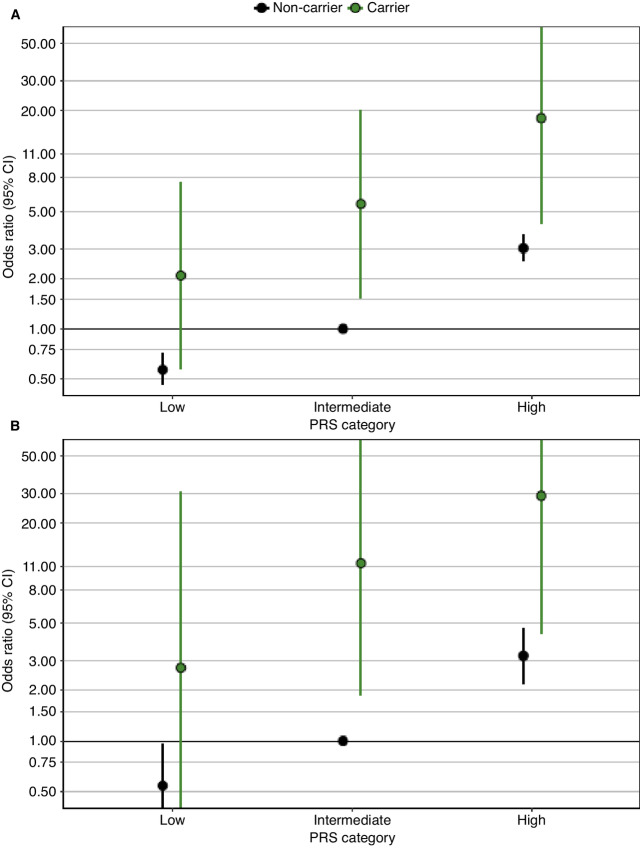
ORs for overall prostate cancer (**A**) and metastatic prostate cancer (**B**) in African ancestry men by PRS and carrier status of rare P/LP/D variants aggregated across *BRCA2, ATM, NBN*, and *PALB2*. The *x*-axis indicates the PRS tertile. The *y*-axis represents OR values for the indicated PRS tertile compared with non-carriers within the intermediate PRS category as the reference group and adjusted by age, study, and the first 10 principal components. Dots represent ORs and error bar lines represent 95% CIs. Black represents results in non-carriers, and green represents results in carriers. Results are also provided in Supplementary Table S6.

The median PRS in controls was similar in MEC (24.48) and UGPCS (24.73) participants (24.57 overall in controls). On average, the PRS was nonsignificantly higher for P/LP/D non-carriers compared with carriers for cases and controls (Supplementary Fig. S1). Only among aggressive cases, was the PRS significantly higher (*P* = 0.036) in non-carriers (mean = 25.25, SD = 0.86) than carriers of P/LP/D variants (mean = 24.97, SD = 0.70; Supplementary Fig. S1; Supplementary Table S7). P/LP/D carrier frequencies were similar in men with a first-degree family history of prostate cancer (0.0% in controls and 2.3% in cases) and without a first-degree family history of prostate cancer (0.7% in controls and 2.6% in cases; Supplementary Table S8). P/LP/D status aggregated across *BRCA2, ATM, NBN,* and *PALB2* was strongly associated with risk of overall prostate cancer (OR = 4.51; 95% CI = 2.18–9.33; *P* = 4.75 × 10^−05^) and metastatic disease (OR = 6.92; 95% CI = 2.34–20.49; *P* = 4.76 × 10^−04^; Supplementary Table S4), as reported previously (5). Compared with men in the intermediate PRS category, men in the high PRS category had increased risk of overall prostate cancer (OR = 3.00; 95% CI = 2.52–3.57; *P* = 2.44 × 10^−35^) and metastatic disease (OR = 3.11; 95% CI = 2.14–4.52; *P* = 2.83 × 10^−09^; Supplementary Table S5). Among P/LP/D non-carriers, the OR for prostate cancer ranged from 0.57 (95% CI = 0.46–0.71; *P* = 2.73 × 10^−7^) in low PRS men to 3.02 (95% CI = 2.53–3.60; *P* = 3.73 × 10^−35^) in high PRS men compared with non-carriers with intermediate PRS (Fig. 1A; Supplementary Table S6). Among P/LP/D carriers, the OR for prostate cancer ranged from 2.08 (95% CI = 0.58–7.49; *P* = 0.26) in low PRS men to 18.06 (95% CI = 4.24–76.84; *P* = 9.01 × 10^−5^) in high PRS men compared with non-carriers with intermediate PRS. When limiting analyses to metastatic cases versus controls, the P/LP/D non-carrier OR ranged from 0.54 (95% CI = 0.31–0.95; *P* = 0.032) in low PRS men to 3.22 (95% CI = 2.20–4.73; *P* = 2.33 × 10^−9^) in high PRS men, while among P/LP/D carriers, the OR ranged from 2.73 (95% CI = 0.24–30.54; *P* = 0.24) in low PRS men to 28.99 (95% CI = 4.39–191.43; *P* = 4.73 × 10^−4^) in high PRS men compared with non-carriers with intermediate PRS (Fig. 1B; Supplementary Table S6). No multiplicative interaction between PRS and P/LP/D carrier status was identified on the basis of the likelihood ratio test and Wald statistic, which suggests an additive effect between the PRS and carrier status (Supplementary Table S9). In analyses stratified by country, results were similar in African American and Ugandan men, although CIs were wide given the reduced sample sizes (Supplementary Tables S10–S15). When repeating analyses for pathogenic variants in four additional genes (*BRCA1, RAD50, MLH1,* and *MSH6*) with weaker evidence of association with prostate cancer risk (5), the risk modifying effect of PRS was similar, albeit the associations were diminished (Supplementary Tables S16 and S17).

**FIGURE 2:**
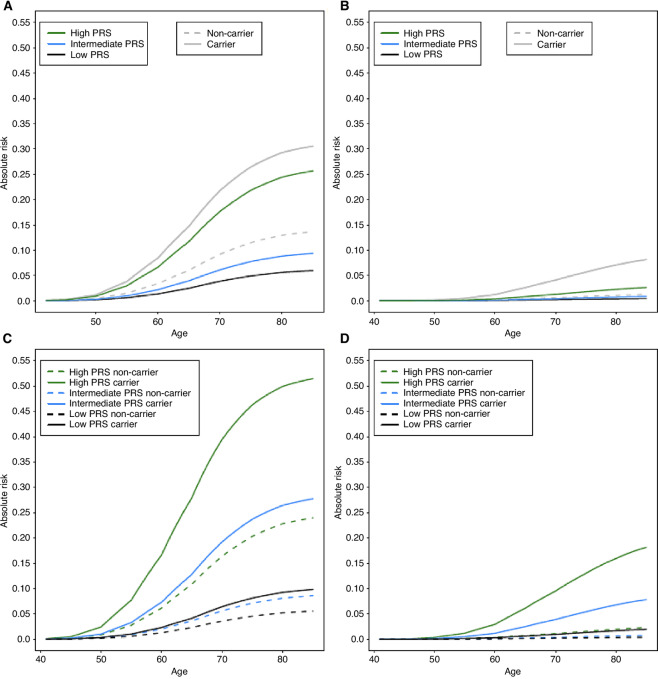
Absolute risk for overall prostate cancer and metastatic prostate cancer in African ancestry men by PRS and carrier status of rare P/LP/D variants in *BRCA2, ATM, NBN*, and *PALB2*. The *x*-axis represents age, and the *y*-axis represents absolute risk. **A,** Absolute risk of overall prostate cancer by age and carrier status (independent of PRS) and PRS category (independent of carrier status). **B,** Absolute risk of metastatic prostate cancer by age and carrier status (independent of PRS) and PRS category (independent of carrier status). For A and B, green lines represent high PRS, blue lines represent intermediate PRS, black lines represent low PRS, solid gray lines represent P/LP/D carriers, and dashed gray lines represent non-carriers. **C,** Absolute risk of overall prostate cancer by age for the combination of carrier status and PRS. **D,** Absolute risk of metastatic prostate cancer by age for the combination of carrier status and PRS. For C and D, solid lines represent P/LP/D carriers, dashed lines represent non-carriers, green lines represent high PRS, blue lines represent intermediate PRS, and black lines represent low PRS. Results are also provided in Supplementary Tables S16–S19.

Absolute risk for prostate cancer by age 85 was 30.5% (95% CI = 10.0–53.7) in P/LP/D carriers (0.6% of the population) and 25.7% (95% CI = 23.3–27.8) in men with high PRS (33% of the population; Fig. 2A; Supplementary Tables S2 and S18). Absolute risk for metastatic prostate cancer by age 85 was 8.2% (95% CI = 0.3–17.8) in P/LP/D carriers and 2.6% (95% CI = 2.2–3.0) in men with high PRS (Fig. 2B; Supplementary Table S19). Considering P/LP/D carrier status and PRS in conjunction, absolute risk of prostate cancer in P/LP/D non-carriers ranged from 5.5% (95% CI = 4.2–7.3) in low PRS men to 23.9% (95% CI = 22.1–25.6) in high PRS men, while among P/LP/D carriers, absolute risk ranged from 9.8% (95% CI = 1.0–33.1) in low PRS men to 51.5% (95% CI = 6.6–87.8) in high PRS men (Fig. 2C; Supplementary Tables S3 and S20). For metastatic disease, the absolute risk in P/LP/D non-carriers ranged from 0.4% (95% CI = 0.2–0.6) in low PRS men to 2.3% (95% CI = 1.8–2.6) in high PRS men, while among P/LP/D carriers, absolute risk ranged from 1.9% (95% CI = 1–15.0) in low PRS men to 18.2% (95% CI = 1–57.1) in high PRS men (Fig. 2D; Supplementary Table S21).

## Discussion

These findings highlight the importance of considering rare and common variants to comprehensively assess prostate cancer risk in men of African ancestry. We found that the association of P/LP/D variants in *BRCA2, ATM, PALB2,* and *NBN* with prostate cancer risk in men of African ancestry varied by a man's PRS profile, with enhanced risk stratification for metastatic and aggressive prostate cancer, suggesting an additive effect of the PRS and carrier status. These findings are highly consistent with what has been reported in studies among men of European ancestry (6) that have examined P/LP/D variants in several high-risk genes (e.g., *HOXB13*, *BRCA2*, *ATM*, and *CHEK2*). In the current study, the set of genes (*ATM*, *BRCA2*, *PALB2*, and *NBN*) were those previously shown to be strongly associated with prostate cancer risk in this African ancestry sample. A similar risk-modifying effect of PRS on carrier status was also observed for genes that were found to have weaker independent associations with overall prostate cancer and aggressive disease (*BRCA1, RAD50, MLH1,* and *MSH6).* While previous larger studies in men of European ancestry permitted finer stratification of PRS (deciles), the smaller sample size of the current study permitted testing only broad PRS strata (tertiles) when examining aggregate effects by carrier status. Despite these differences, the trends between studies when examining this question were very similar. The higher PRS observed in non-carriers than carriers of P/LP/D variants also suggests independent and separate processes involved in prostate cancer development.

We estimate that absolute risk of prostate cancer for men who carry P/LP/D variants is heavily influenced by PRS status. At age 60, absolute risk ranges from 2% for men with a low PRS to 16% for men with a high PRS for men who carry P/LP/D variants; by age 70 these estimates range from 6% to 40%, respectively (Supplementary Table S18). While *BRCA2* carriers are recommended to initiate prostate cancer screening at age 40 or 10 years prior to the youngest prostate cancer diagnosis in a family (with consideration for *BRCA1, ATM, HOXB13*, and mismatch repair gene carriers; ref. 4), our findings in African ancestry men and others in European ancestry men (6, 26–29) suggest that PRS should also be incorporated into prostate cancer risk assessment to inform age-based screening guidelines. Future studies with larger sample sizes of diverse African ancestry men will allow for finer PRS subgroups to be examined together with rare variant carrier status for more accurate estimation of genetic risk for overall prostate cancer, as well as metastatic and aggressive prostate cancer across the African diaspora.

## Supplementary Material

Supplementary Figure 1 PRS distribution by carrier status of P/LP/D variants in BRCA2, ATM, PALB2, and NBN and prostate cancer status.

Supplementary Table 1 Rare germline pathogenic, likely pathogenic, or deleterious (P/LP/D) variants (n=51) in BRCA2, ATM, NBN, and PALB2 identified among men of African ancestry.

Supplementary Table 2 Weighted independent effect of PRS and P/LP/D variants in BRCA2, ATM, NBN, and PALB2 on PCa risk in African ancestry men.

Supplementary Table 3 Weighted aggregate effects of PRS and P/LP/D variants in BRCA2, ATM, NBN, and PALB2 on PCa risk in African ancestry men.

Supplementary Table 4 Aggregate effect of P/LP/D carrier status across BRCA2, ATM, NBN, and PALB2 genes on PCa risk in African ancestry men.

Supplementary Table 5 Association of PRS and PCa risk in African ancestry men.

Supplementary Table 6 Aggregate effect of PRS and P/LP/D variants in BRCA2, ATM, NBN, and PALB2 on PCa risk in African ancestry men.

Supplementary Table 7 Carrier frequency of P/LP/D variants in BRCA2, ATM, NBN, and PALB2 by PRS category in African ancestry men.

Supplementary Table 8 Carrier frequency of P/LP/D variants in BRCA2, ATM, NBN, and PALB2 by family history in African ancestry men.

Supplementary Table 9 Interaction effect of PRS and P/LP/D carrier status across BRCA2, ATM, NBN, and PALB2 genes on PCa risk in African ancestry men. LRT: Likelihood ratio test.

Supplementary Table 10 Aggregate effect of P/LP/D carrier status across BRCA2, ATM, NBN, and PALB2 genes on PCa risk in African American men.

Supplementary Table 11 Association of PRS and PCa risk in African American men.

Supplementary Table 12 Aggregate effect of PRS and P/LP/D variants in BRCA2, ATM, NBN, and PALB2 on PCa risk in African American men.

Supplementary Table 13 Aggregate effect of P/LP/D carrier status across BRCA2, ATM, NBN, and PALB2 genes on PCa risk in Ugandan men.

Supplementary Table 14 Association of PRS and PCa risk in Ugandan men.

Supplementary Table 15 Aggregate effect of PRS and P/LP/D variants in BRCA2, ATM, NBN, and PALB2 on PCa risk in Ugandan men.

Supplementary Table 16 Aggregate effect of P/LP/D carrier status across BRCA1, RAD50, MLH1, and MSH6 genes on PCa risk in African ancestry men.

Supplementary Table 17 Aggregate effect of PRS and P/LP/D variants in BRCA1, RAD50, MLH1, and MSH6 on PCa risk in African ancestry men.

Supplementary Table 18 Absolute risk of PCa by PRS and P/LP/D variants in BRCA2, ATM, NBN, and PALB2 independently in African ancestry men.

Supplementary Table 19 Absolute risk of metastatic PCa by PRS and P/LP/D variants in BRCA2, ATM, NBN, and PALB2 independently in African ancestry men.

Supplementary Table 20 Absolute risk of PCa by PRS and P/LP/D variants in BRCA2, ATM, NBN, and PALB2 combined in African ancestry men.

Supplementary Table 21 Absolute risk of metastatic PCa by PRS and P/LP/D variants in BRCA2, ATM, NBN, and PALB2 combined in African ancestry men.
