# Supplementary material for: Polygenic Risk Score Modifies Prostate Cancer Risk of Pathogenic Variants in Men of African Ancestry
Source: Cancer Res Commun. 2023 Dec 14;3(12):2544–50. doi: 10.1158/2767-9764.CRC-23-0022 (PMC10720390; doi:10.1158/2767-9764.CRC-23-0022)
Supplement: Supplementary Figure 1 — PRS distribution by carrier status of P/LP/D variants in BRCA2, ATM, PALB2, and NBN and prostate cancer status. [file crc-23-0022-s01.docx]

**
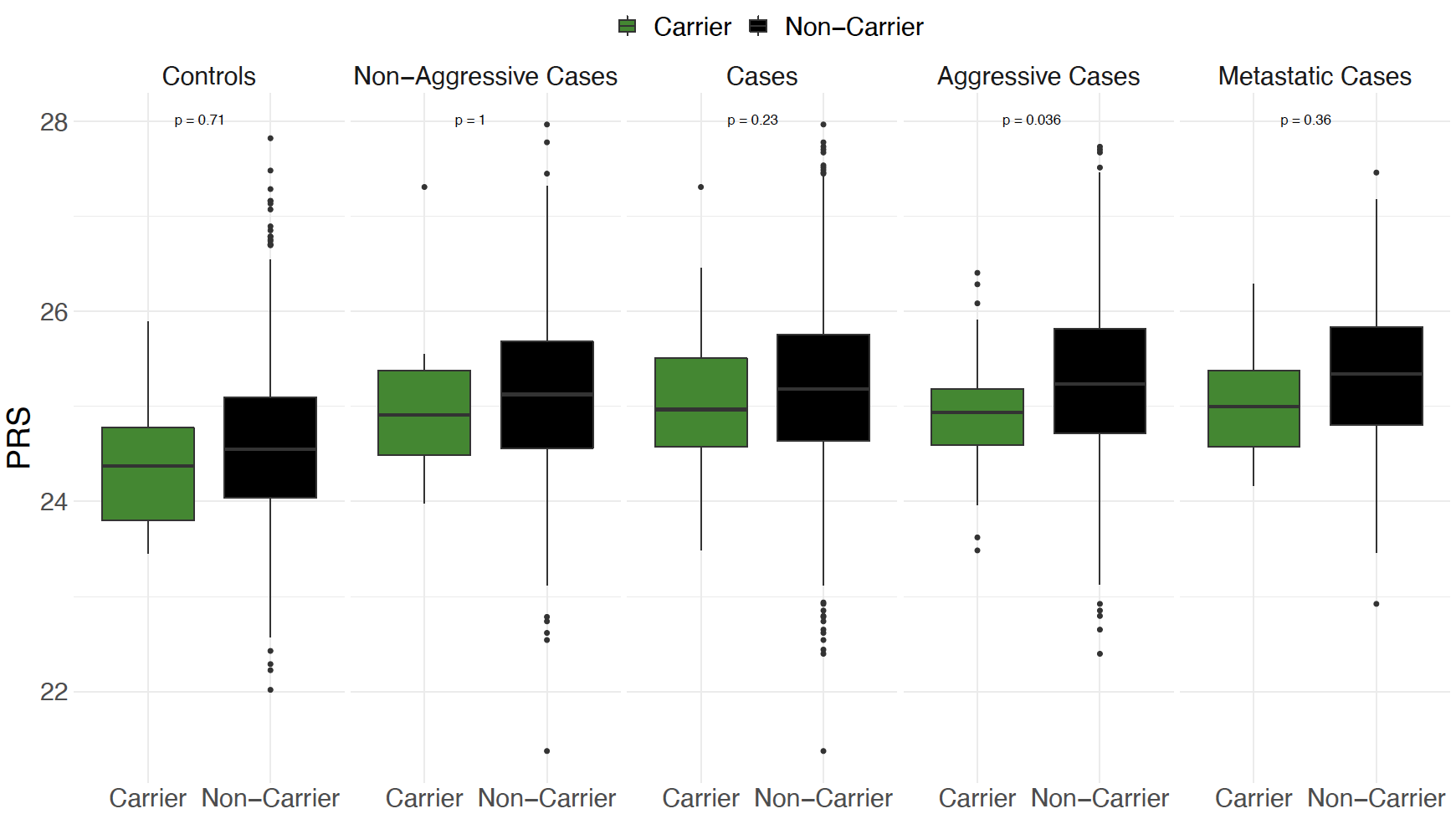
**

**Supplementary Figure 1**. PRS distribution by carrier status of P/LP/D variants in *BRCA2*, *ATM*, *PALB2*, and *NBN* and prostate cancer status. PRS difference between carriers and non-carriers was calculated using a two-sided t-test.
