# Supplementary material for: Polygenic Risk Score Modifies Prostate Cancer Risk of Pathogenic Variants in Men of African Ancestry
Source: Cancer Res Commun. 2023 Dec 14;3(12):2544–50. doi: 10.1158/2767-9764.CRC-23-0022 (PMC10720390; doi:10.1158/2767-9764.CRC-23-0022)
Supplement: Supplementary Table 1 — Rare germline pathogenic, likely pathogenic, or deleterious (P/LP/D) variants (n=51) in BRCA2, ATM, NBN, and PALB2 identified among men of African ancestry. [file crc-23-0022-s02.docx]

**Supplementary Table 1**. Rare germline pathogenic, likely pathogenic, or deleterious (P/LP/D) variants (n=51) in *BRCA2, ATM, NBN,* and *PALB2* identified among men of African ancestry.

| **Gene** | **dbSNP^1^** | **Chromosome** | **Position** | **Allele change^2^** | **Base change amino acid change** | **Frequency^3^** |
| --- | --- | --- | --- | --- | --- | --- |
| *ATM* | . | 11 | 108121559 | T>A | c.1367T>A_p.Leu456X | 0.0001 |
| *ATM* | . | 11 | 108124551 | CA>C | c.1914delA | 0.0001 |
| *ATM* | rs777849257 | 11 | 108124665 | C>T | c.2023C>T_p.Gln675X | 0.0001 |
| *ATM* | rs587781558 | 11 | 108141874 | G>A | c.2921+1G>A | 0.0001 |
| *ATM* | rs587779833 | 11 | 108150305 | C>G | c.3372C>G_p.Tyr1124X | 0.0001 |
| *ATM* | . | 11 | 108170500 | C>T | c.5065C>T_p.Gln1689X | 0.0001 |
| *ATM* | rs878853525 | 11 | 108175454 | T>A | c.5549T>A_p.Leu1850X | 0.0001 |
| *ATM* | . | 11 | 108196843 | C>CT | c.6867dupT | 0.0001 |
| *ATM* | . | 11 | 108200998 | GA>G | c.7368delA | 0.0001 |
| *ATM* | . | 11 | 108202268 | A>AT | c.7614dupT | 0.0001 |
| *ATM* | rs377349459 | 11 | 108203613 | G>A | c.7913G>A_p.Trp2638X | 0.0001 |
| *ATM* | rs587779844 | 11 | 108172425 | C>T | c.5228C>T_p.Thr1743Ile | 0.0001 |
| *ATM* | rs202206540 | 11 | 108186796 | G>A | c.6154G>A_p.Glu2052Lys | 0.0001 |
| *ATM* | rs730881384 | 11 | 108204682 | C>A | c.7997C>A_p.Thr2666Asn | 0.0001 |
| *ATM* | rs587782292 | 11 | 108236086 | C>T | c.9022C>T_p.Arg3008Cys | 0.0001 |
| *ATM* | rs786203421 | 11 | 108198391 | TTACA>T | c.7000_7003delTACA | 0.0003 |
| *BRCA2* | . | 13 | 32906663 | GA>G | c.1053delA | 0.0001 |
| *BRCA2* | rs397507265 | 13 | 32906818 | AG>A | c.1205delG | 0.0001 |
| *BRCA2* | rs80358464 | 13 | 32907415 | T>A | c.1800T>A_p.Tyr600X | 0.0001 |
| *BRCA2* | rs397507279 | 13 | 32910462 | T>A | c.1970T>A_p.Leu657X | 0.0001 |
| *BRCA2* | rs398122753 | 13 | 32911297 | TA>T | c.2808delA | 0.0001 |
| *BRCA2* | rs886038093 | 13 | 32912210 | CTGTT>C | c.3720_3723delGTTT | 0.0001 |
| *BRCA2* | rs397507323 | 13 | 32912618 | GGAAA>G | c.4127_4130delGAAA | 0.0001 |
| *BRCA2* | rs80359464 | 13 | 32913199 | CAG>C | c.4712_4713delAG | 0.0001 |
| *BRCA2* | . | 13 | 32913965 | G>GCAGC | c.5476_5479dupGCCA | 0.0001 |
| *BRCA2* | rs80359535 | 13 | 32914260 | ACATT>A | c.5771_5774delTTCA | 0.0001 |
| *BRCA2* | rs80359550 | 13 | 32914437 | GT>G | c.5946delT | 0.0001 |
| *BRCA2* | rs80358851 | 13 | 32914616 | C>T | c.6124C>T_p.Gln2042X | 0.0001 |
| *BRCA2* | rs80358928 | 13 | 32929014 | C>T | c.7024C>T_p.Gln2342X | 0.0001 |
| *BRCA2* | rs80359641 | 13 | 32929192 | GACCA>G | c.7208_7211delCCAA | 0.0001 |
| *BRCA2* | . | 13 | 32953452 | A>G | c.8755-2A>G | 0.0001 |
| *BRCA2* | . | 13 | 32953596 | T>TCACAACC | c.8898_8904dup7 | 0.0001 |
| *BRCA2* | rs80359149 | 13 | 32953903 | G>A | c.8970G>A_p.Trp2990X | 0.0001 |
| *BRCA2* | rs80359752 | 13 | 32954272 | G>GA | c.9253dupA | 0.0001 |
| *BRCA2* | rs80359212 | 13 | 32968951 | C>T | c.9382C>T_p.Arg3128X | 0.0001 |
| *BRCA2* | . | 13 | 32972342 | C>A | c.9692C>A_p.Ser3231X | 0.0001 |
| *BRCA2* | rs730881619 | 13 | 32972540 | C>CATTT | c.9891_9894dupATTT | 0.0001 |
| *BRCA2* | rs80359412 | 13 | 32912353 | TAATA>T | c.3865_3868delAAAT | 0.0003 |
| *BRCA2* | . | 13 | 32914123 | C>CA | c.5633dupA | 0.0003 |
| *NBN* | rs786201965 | 8 | 90955595 | C>T | c.2071-1G>A | 0.0001 |
| *NBN* | rs764884516 | 8 | 90965832 | AG>A | c.1484delC | 0.0001 |
| *NBN* | rs864622090 | 8 | 90976737 | T>A | c.897-2A>T | 0.0001 |
| *NBN* | rs751567476 | 8 | 90990553 | T>C | c.481-2A>G | 0.0001 |
| *NBN* | rs786202494 | 8 | 90993711 | TTA>T | c.210_211delTA | 0.0001 |
| *NBN* | rs587781891 | 8 | 90994997 | TG>T | c.123delC | 0.0001 |
| *NBN* | . | 8 | 90955565 | TG>T | c.2099delC | 0.0003 |
| *NBN* | . | 8 | 90993748 | G>A | c.175C>T_p.Gln59X | 0.0004 |
| *PALB2* | rs180177133 | 16 | 23625409 | AT>A | c.3116delA | 0.0001 |
| *PALB2* | rs180177132 | 16 | 23632683 | C>T | c.3113G>A_p.Trp1038X | 0.0001 |
| *PALB2* | rs747148023 | 16 | 23641062 | CAG>C | c.2411_2412delCT | 0.0001 |
| *PALB2* | rs180177110 | 16 | 23641218 | G>A | c.2257C>T_p.Arg753X | 0.0001 |

^1^dbSNP identifiers retrieved from the National Center for Biotechnology Information (NCBI); based on human genome build 37 (GRCh37)

^2^Common allele>rare allele

^3^Allele frequency based on the complete targeted sequencing sample of 3,579 men [Matejcic et al, JCO Precision Oncology, 2020]
