# Supplementary material for: Polygenic Risk Score Modifies Prostate Cancer Risk of Pathogenic Variants in Men of African Ancestry
Source: Cancer Res Commun. 2023 Dec 14;3(12):2544–50. doi: 10.1158/2767-9764.CRC-23-0022 (PMC10720390; doi:10.1158/2767-9764.CRC-23-0022)
Supplement: Supplementary Table 3 — Weighted aggregate effects of PRS and P/LP/D variants in BRCA2, ATM, NBN, and PALB2 on PCa risk in African ancestry men. [file crc-23-0022-s04.docx]

**Supplementary Table 3.** Weighted aggregate effects of PRS and P/LP/D variants in *BRCA2*, *ATM*, *NBN*, and *PALB2* on PCa risk in African ancestry men. The weighted PCa ORs weight the overall PCa and aggressive PCa ORs in Supplementary Table 6 based on the number of expected aggressive cases in a general population.

| **PRS Category** | **Carrier Status** | **Weighted OR** |
| --- | --- | --- |
| Low PRS | Non-Carrier | 0.63 |
| Low PRS | Carrier | 1.15 |
| Intermediate PRS | Non-Carrier | Ref |
| Intermediate PRS | Carrier | 3.75 |
| High PRS | Non-Carrier | 3.14 |
| High PRS | Carrier | 9.05 |
