# Supplementary material for: Polygenic Risk Score Modifies Prostate Cancer Risk of Pathogenic Variants in Men of African Ancestry
Source: Cancer Res Commun. 2023 Dec 14;3(12):2544–50. doi: 10.1158/2767-9764.CRC-23-0022 (PMC10720390; doi:10.1158/2767-9764.CRC-23-0022)
Supplement: Supplementary Table 4 — Aggregate effect of P/LP/D carrier status across BRCA2, ATM, NBN, and PALB2 genes on PCa risk in African ancestry men. [file crc-23-0022-s05.docx]

**Supplementary Table 4.** Aggregate effect of P/LP/D carrier status across *BRCA2*, *ATM*, *NBN*, and *PALB2* genes on PCa risk in African ancestry men.

|  | **Carrier Status** | **N Controls** | **N Cases** | **OR** | **95% CI** | **P value** |
| --- | --- | --- | --- | --- | --- | --- |
| **Overall PCa**  **versus controls** | Non-Carrier | 1,415 | 1,752 | Ref | -- | -- |
|  | Carrier | 9 | 44 | 4.51 | 2.18 to 9.33 | 4.75x10^-5^ |
| **Metastatic PCa**  **versus controls** | Non-Carrier | 1415 | 213 | Ref | -- | -- |
|  | Carrier | 9 | 9 | 6.92 | 2.34 to 20.49 | 0.001 |
| **Aggressive PCa**  **versus controls** | Non-Carrier | 1,415 | 873 | Ref | -- | -- |
|  | Carrier | 9 | 30 | 5.91 | 2.77 to 12.63 | 4.36x10^-6^ |
| **Non-aggressive PCa**  **versus controls** | Non-Carrier | 1,415 | 727 | Ref | -- | -- |
|  | Carrier | 9 | 8 | 1.58 | 0.58 to 4.34 | 0.380 |
