# Supplementary material for: Polygenic Risk Score Modifies Prostate Cancer Risk of Pathogenic Variants in Men of African Ancestry
Source: Cancer Res Commun. 2023 Dec 14;3(12):2544–50. doi: 10.1158/2767-9764.CRC-23-0022 (PMC10720390; doi:10.1158/2767-9764.CRC-23-0022)
Supplement: Supplementary Table 5 — Association of PRS and PCa risk in African ancestry men. [file crc-23-0022-s06.docx]

**Supplementary Table 5.** Association of PRS and PCa risk in African ancestry men.

|  | **PRS Category** | **N Controls** | **N Cases** | **OR** | **95% CI** | **P value** |
| --- | --- | --- | --- | --- | --- | --- |
| **Overall PCa**  **versus controls** | Low PRS | 475 | 228 | 0.57 | 0.46 to 0.70 | 1.47x10^-7^ |
|  | Intermediate PRS | 474 | 391 | Ref | -- | -- |
|  | High PRS | 475 | 1,177 | 3.00 | 2.52 to 3.57 | 2.44x10^-35^ |
| **Metastatic PCa**  **versus controls** | Low PRS | 475 | 21 | 0.53 | 0.30 to 0.91 | 0.022 |
|  | Intermediate PRS | 474 | 44 | Ref | -- | -- |
|  | High PRS | 475 | 157 | 3.11 | 2.14 to 4.52 | 2.83x10^-9^ |
| **Aggressive PCa**  **versus controls** | Low PRS | 475 | 101 | 0.53 | 0.40 to 0.70 | 3.96x10^-6^ |
|  | Intermediate PRS | 474 | 192 | Ref | -- | -- |
|  | High PRS | 475 | 610 | 3.14 | 2.54 to 3.86 | 8.59x10^-27^ |
| **Non-aggressive PCa**  **versus controls** | Low PRS | 475 | 113 | 0.69 | 0.52 to 0.92 | 0.012 |
|  | Intermediate PRS | 474 | 161 | Ref | -- | -- |
|  | High PRS | 475 | 461 | 3.10 | 2.43 to 3.95 | 6.11x10^-20^ |
