# Supplementary material for: Polygenic Risk Score Modifies Prostate Cancer Risk of Pathogenic Variants in Men of African Ancestry
Source: Cancer Res Commun. 2023 Dec 14;3(12):2544–50. doi: 10.1158/2767-9764.CRC-23-0022 (PMC10720390; doi:10.1158/2767-9764.CRC-23-0022)
Supplement: Supplementary Table 6 — Aggregate effect of PRS and P/LP/D variants in BRCA2, ATM, NBN, and PALB2 on PCa risk in African ancestry men. [file crc-23-0022-s07.docx]

**Supplementary Table 6.** Aggregate effect of PRS and P/LP/D variants in *BRCA2*, *ATM*, *NBN*, and *PALB2* on PCa risk in African ancestry men.

|  | **PRS Category** | **Carrier Status** | **N Controls** | **N Cases** | **OR** | **95% CI** | **P value** |
| --- | --- | --- | --- | --- | --- | --- | --- |
| **Overall PCa**  **versus controls** | Low PRS | Non-Carrier | 471 | 222 | 0.57 | 0.47 to 0.71 | 2.73x10^-7^ |
|  | Low PRS | Carrier | 4 | 6 | 2.08 | 0.58 to 7.49 | 0.262 |
|  | Intermediate PRS | Non-Carrier | 471 | 379 | Ref | -- | -- |
|  | Intermediate PRS | Carrier | 3 | 12 | 5.56 | 1.54 to 20.02 | 0.009 |
|  | High PRS | Non-Carrier | 473 | 1,151 | 3.02 | 2.53 to 3.60 | 3.73x10^-35^ |
|  | High PRS | Carrier | 2 | 26 | 18.06 | 4.24 to 76.84 | 9.01x10^-5^ |
| **Metastatic PCa**  **versus controls** | Low PRS | Non-Carrier | 471 | 20 | 0.54 | 0.31 to 0.95 | 0.032 |
|  | Low PRS | Carrier | 4 | 1 | 2.73 | 0.24 to 30.54 | 0.415 |
|  | Intermediate PRS | Non-Carrier | 471 | 41 | Ref | -- | -- |
|  | Intermediate PRS | Carrier | 3 | 3 | 11.47 | 1.87 to 70.18 | 0.008 |
|  | High PRS | Non-Carrier | 473 | 152 | 3.22 | 2.20 to 4.73 | 2.33x10^-9^ |
|  | High PRS | Carrier | 2 | 5 | 28.99 | 4.39 to 191.43 | 0.001 |
| **Aggressive PCa**  **versus controls** | Low PRS | Non-Carrier | 471 | 97 | 0.53 | 0.40 to 0.70 | 6.24x10^-6^ |
|  | Low PRS | Carrier | 4 | 4 | 2.97 | 0.73 to 12.16 | 0.129 |
|  | Intermediate PRS | Non-Carrier | 471 | 184 | Ref | -- | -- |
|  | Intermediate PRS | Carrier | 3 | 8 | 7.08 | 1.84 to 27.19 | 0.004 |
|  | High PRS | Non-Carrier | 473 | 592 | 3.16 | 2.56 to 3.91 | 1.62x10^-26^ |
|  | High PRS | Carrier | 2 | 18 | 23.58 | 5.39 to 103.20 | 2.72x10^-5^ |
| **Non-aggressive PCa versus controls** | Low PRS | Non-Carrier | 471 | 112 | 0.70 | 0.53 to 0.94 | 0.016 |
|  | Low PRS | Carrier | 4 | 1 | 0.66 | 0.07 to 6.37 | 0.716 |
|  | Intermediate PRS | Non-Carrier | 471 | 158 | Ref | -- | -- |
|  | Intermediate PRS | Carrier | 3 | 3 | 2.59 | 0.51 to 13.27 | 0.252 |
|  | High PRS | Non-Carrier | 473 | 457 | 3.13 | 2.45 to 3.99 | 5.44x10^-20^ |
|  | High PRS | Carrier | 2 | 4 | 5.16 | 0.76 to 35.17 | 0.094 |
