# Supplementary material for: Polygenic Risk Score Modifies Prostate Cancer Risk of Pathogenic Variants in Men of African Ancestry
Source: Cancer Res Commun. 2023 Dec 14;3(12):2544–50. doi: 10.1158/2767-9764.CRC-23-0022 (PMC10720390; doi:10.1158/2767-9764.CRC-23-0022)
Supplement: Supplementary Table 7 — Carrier frequency of P/LP/D variants in BRCA2, ATM, NBN, and PALB2 by PRS category in African ancestry men. [file crc-23-0022-s08.docx]

**Supplementary Table 7.** Carrier frequency of P/LP/D variants in *BRCA2*, *ATM*, *NBN*, and *PALB2* by PRS category in African ancestry men.

| **PRS Category** | **Participant Status** | **N** | **N Carriers** | **Carrier Frequency** |
| --- | --- | --- | --- | --- |
| Low PRS | Controls | 475 | 4 | 0.8% |
| Low PRS | Cases | 228 | 6 | 2.6% |
| Low PRS | Aggressive Cases | 101 | 4 | 4.0% |
| Low PRS | Non-Aggressive Cases | 113 | 1 | 0.9% |
| Low PRS | Metastatic Cases | 21 | 1 | 4.8% |
| Intermediate PRS | Controls | 474 | 3 | 0.6% |
| Intermediate PRS | Cases | 391 | 12 | 3.1% |
| Intermediate PRS | Aggressive Cases | 192 | 8 | 4.2% |
| Intermediate PRS | Non-Aggressive Cases | 161 | 3 | 1.9% |
| Intermediate PRS | Metastatic Cases | 44 | 3 | 6.8% |
| High PRS | Controls | 475 | 2 | 0.4% |
| High PRS | Cases | 1177 | 26 | 2.2% |
| High PRS | Aggressive Cases | 610 | 18 | 3.0% |
| High PRS | Non-Aggressive Cases | 461 | 4 | 0.9% |
| High PRS | Metastatic Cases | 157 | 5 | 3.2% |
