# Supplementary material for: Polygenic Risk Score Modifies Prostate Cancer Risk of Pathogenic Variants in Men of African Ancestry
Source: Cancer Res Commun. 2023 Dec 14;3(12):2544–50. doi: 10.1158/2767-9764.CRC-23-0022 (PMC10720390; doi:10.1158/2767-9764.CRC-23-0022)
Supplement: Supplementary Table 8 — Carrier frequency of P/LP/D variants in BRCA2, ATM, NBN, and PALB2 by family history in African ancestry men. [file crc-23-0022-s09.docx]

**Supplementary Table 8.** Carrier frequency of P/LP/D variants in *BRCA2*, *ATM*, *NBN*, and *PALB2* by family history in African ancestry men.

| **Family History of PCa Status** | **Participant Status** | **N** | **N Carriers** | **Carrier Frequency** |
| --- | --- | --- | --- | --- |
| **No First-degree family history of PCa** | Controls | 1190 | 8 | 0.7% |
|  | Cases | 1249 | 32 | 2.6% |
| **First-degree family history of PCa** | Controls | 107 | 0 | 0.0% |
|  | Cases | 308 | 7 | 2.3% |
