# Supplementary material for: Polygenic Risk Score Modifies Prostate Cancer Risk of Pathogenic Variants in Men of African Ancestry
Source: Cancer Res Commun. 2023 Dec 14;3(12):2544–50. doi: 10.1158/2767-9764.CRC-23-0022 (PMC10720390; doi:10.1158/2767-9764.CRC-23-0022)
Supplement: Supplementary Table 9 — Interaction effect of PRS and P/LP/D carrier status across BRCA2, ATM, NBN, and PALB2 genes on PCa risk in African ancestry men. LRT: Likelihood ratio test. [file crc-23-0022-s10.docx]

**Supplementary Table 9.** Interaction effect of PRS and P/LP/D carrier status across *BRCA2*, *ATM*, *NBN*, and *PALB2* genes on PCa risk in African ancestry men. LRT: Likelihood ratio test.

|  | **OR** | **95% CI** | **Wald P value** | **LRT P value** |
| --- | --- | --- | --- | --- |
| **Overall PCa**  **versus controls** | 1.25 | 0.40 to 3.91 | 0.695 | 0.690 |
| **Metastatic PCa**  **versus controls** | 1.15 | 0.24 to 5.60 | 0.863 | 0.861 |
| **Aggressive PCa**  **versus control** | 0.95 | 0.30 to 3.01 | 0.928 | 0.928 |
| **Non-aggressive PCa**  **versus controls** | 1.15 | 0.28 to 4.73 | 0.847 | 0.844 |
