# Supplementary material for: Polygenic Risk Score Modifies Prostate Cancer Risk of Pathogenic Variants in Men of African Ancestry
Source: Cancer Res Commun. 2023 Dec 14;3(12):2544–50. doi: 10.1158/2767-9764.CRC-23-0022 (PMC10720390; doi:10.1158/2767-9764.CRC-23-0022)
Supplement: Supplementary Table 10 — Aggregate effect of P/LP/D carrier status across BRCA2, ATM, NBN, and PALB2 genes on PCa risk in African American men. [file crc-23-0022-s11.docx]

**Supplementary Table 10.** Aggregate effect of P/LP/D carrier status across *BRCA2*, *ATM*, *NBN*, and *PALB2* genes on PCa risk in African American men.

|  | **Carrier Status** | **N Controls** | **N Cases** | **OR** | **95% CI** | **P value** |
| --- | --- | --- | --- | --- | --- | --- |
| **Overall PCa**  **versus controls** | Non-Carrier | 958 | 1264 | Ref | -- | -- |
|  | Carrier | 7 | 22 | 2.11 | 0.79 to 5.63 | 0.135 |
| **Metastatic PCa**  **versus controls** | Non-Carrier | 958 | 69 | Ref | -- | -- |
|  | Carrier | 7 | 1 | NA | NA | NA |
| **Aggressive PCa**  **versus controls** | Non-Carrier | 958 | 577 | Ref | -- | -- |
|  | Carrier | 7 | 14 | 2.80 | 0.91 to 8.61 | 0.072 |
| **Non-aggressive PCa**  **versus controls** | Non-Carrier | 958 | 687 | Ref | -- | -- |
|  | Carrier | 7 | 8 | 1.43 | 0.41 to 4.97 | 0.571 |
