# Supplementary material for: Polygenic Risk Score Modifies Prostate Cancer Risk of Pathogenic Variants in Men of African Ancestry
Source: Cancer Res Commun. 2023 Dec 14;3(12):2544–50. doi: 10.1158/2767-9764.CRC-23-0022 (PMC10720390; doi:10.1158/2767-9764.CRC-23-0022)
Supplement: Supplementary Table 11 — Association of PRS and PCa risk in African American men. [file crc-23-0022-s12.docx]

**Supplementary Table 11.** Association of PRS and PCa risk in African American men.

|  | **PRS Category** | **N Controls** | **N Cases** | **OR** | **95% CI** | **P value** |
| --- | --- | --- | --- | --- | --- | --- |
| **Overall PCa**  **versus controls** | Low PRS | 355 | 187 | 0.70 | 0.53 to 0.93 | 0.013 |
|  | Intermediate PRS | 331 | 275 | Ref | -- | -- |
|  | High PRS | 279 | 824 | 3.10 | 2.43 to 3.95 | 7.16x10^-20^ |
| **Metastatic PCa**  **versus controls** | Low PRS | 355 | 9 | 0.53 | 0.17 to 1.63 | 0.269 |
|  | Intermediate PRS | 331 | 14 | Ref | -- | -- |
|  | High PRS | 279 | 47 | 2.22 | 0.96 to 5.16 | 0.064 |
| **Aggressive PCa**  **versus controls** | Low PRS | 355 | 79 | 0.58 | 0.40 to 0.85 | 0.005 |
|  | Intermediate PRS | 331 | 124 | Ref | -- | -- |
|  | High PRS | 279 | 388 | 2.85 | 2.10 to 3.88 | 2.13x10^-11^ |
| **Non-aggressive PCa**  **versus controls** | Low PRS | 355 | 108 | 0.81 | 0.57 to 1.15 | 0.244 |
|  | Intermediate PRS | 331 | 151 | Ref | -- | -- |
|  | High PRS | 279 | 436 | 3.37 | 2.50 to 4.55 | 1.97x10^-15^ |
