# Supplementary material for: Polygenic Risk Score Modifies Prostate Cancer Risk of Pathogenic Variants in Men of African Ancestry
Source: Cancer Res Commun. 2023 Dec 14;3(12):2544–50. doi: 10.1158/2767-9764.CRC-23-0022 (PMC10720390; doi:10.1158/2767-9764.CRC-23-0022)
Supplement: Supplementary Table 12 — Aggregate effect of PRS and P/LP/D variants in BRCA2, ATM, NBN, and PALB2 on PCa risk in African American men. [file crc-23-0022-s13.docx]

**Supplementary Table 12.** Aggregate effect of PRS and P/LP/D variants in *BRCA2*, *ATM*, *NBN*, and *PALB2* on PCa risk in African American men.

|  | **PRS Category** | **Carrier Status** | **N Controls** | **N Cases** | **OR** | **95% CI** | **P value** |
| --- | --- | --- | --- | --- | --- | --- | --- |
| **Overall PCa**  **versus controls** | Low PRS | Non-Carrier | 352 | 183 | 0.71 | 0.54 to 0.94 | 0.018 |
|  | Low PRS | Carrier | 3 | 4 | 0.80 | 0.08 to 7.82 | 0.846 |
|  | Intermediate PRS | Non-Carrier | 328 | 268 | Ref | -- | -- |
|  | Intermediate PRS | Carrier | 3 | 7 | 2.69 | 0.59 to 12.23 | 0.201 |
|  | High PRS | Non-Carrier | 278 | 813 | 3.11 | 2.44 to 3.98 | 8.15x10^-20^ |
|  | High PRS | Carrier | 1 | 11 | 9.85 | 1.13 to 85.91 | 0.038 |
| **Metastatic PCa**  **versus controls** | Low PRS | Non-Carrier | 352 | 9 | 0.53 | 0.17 to 1.62 | 0.266 |
|  | Low PRS | Carrier | 3 | 0 | NA | NA | NA |
|  | Intermediate PRS | Non-Carrier | 328 | 14 | Ref | -- | -- |
|  | Intermediate PRS | Carrier | 3 | 0 | NA | NA | NA |
|  | High PRS | Non-Carrier | 278 | 46 | 2.21 | 0.95 to 5.13 | 0.065 |
|  | High PRS | Carrier | 1 | 1 | NA | NA | NA |
| **Aggressive PCa**  **versus controls** | Low PRS | Non-Carrier | 352 | 76 | 0.58 | 0.40 to 0.86 | 0.006 |
|  | Low PRS | Carrier | 3 | 3 | 1.77 | 0.18 to 17.82 | 0.630 |
|  | Intermediate PRS | Non-Carrier | 328 | 120 | Ref | -- | -- |
|  | Intermediate PRS | Carrier | 3 | 4 | 2.81 | 0.46 to 17.31 | 0.266 |
|  | High PRS | Non-Carrier | 278 | 381 | 2.86 | 2.10 to 3.90 | 2.74x10^-11^ |
|  | High PRS | Carrier | 1 | 7 | 10.13 | 1.02 to 100.94 | 0.048 |
| **Non-aggressive PCa versus controls** | Low PRS | Non-Carrier | 352 | 107 | 0.83 | 0.59 to 1.18 | 0.301 |
|  | Low PRS | Carrier | 3 | 1 | NA | NA | NA |
|  | Intermediate PRS | Non-Carrier | 328 | 148 | Ref | -- | -- |
|  | Intermediate PRS | Carrier | 3 | 3 | 2.39 | 0.39 to 14.82 | 0.348 |
|  | High PRS | Non-Carrier | 278 | 432 | 3.40 | 2.51 to 4.60 | 2.11x10^-15^ |
|  | High PRS | Carrier | 1 | 4 | 7.85 | 0.69 to 89.48 | 0.097 |
