# Supplementary material for: Polygenic Risk Score Modifies Prostate Cancer Risk of Pathogenic Variants in Men of African Ancestry
Source: Cancer Res Commun. 2023 Dec 14;3(12):2544–50. doi: 10.1158/2767-9764.CRC-23-0022 (PMC10720390; doi:10.1158/2767-9764.CRC-23-0022)
Supplement: Supplementary Table 13 — Aggregate effect of P/LP/D carrier status across BRCA2, ATM, NBN, and PALB2 genes on PCa risk in Ugandan men. [file crc-23-0022-s14.docx]

**Supplementary Table 13.** Aggregate effect of P/LP/D carrier status across *BRCA2*, *ATM*, *NBN*, and *PALB2* genes on PCa risk in Ugandan men.

|  | **Carrier Status** | **N Controls** | **N Cases** | **OR** | **95% CI** | **P value** |
| --- | --- | --- | --- | --- | --- | --- |
| **Overall PCa**  **versus controls** | Non-Carrier | 457 | 488 | Ref | -- | -- |
|  | Carrier | 2 | 22 | 11.30 | 2.54 to 50.22 | 0.001 |
| **Metastatic PCa**  **versus controls** | Non-Carrier | 457 | 144 | Ref | -- | -- |
|  | Carrier | 2 | 8 | 10.91 | 2.08 to 57.14 | 0.005 |
| **Aggressive PCa**  **versus controls** | Non-Carrier | 457 | 296 | Ref | -- | -- |
|  | Carrier | 2 | 16 | 10.67 | 2.33 to 48.73 | 0.002 |
| **Non-aggressive PCa**  **versus controls** | Non-Carrier | 457 | 40 | Ref | -- | -- |
|  | Carrier | 2 | 0 | NA | NA | NA |
