# Supplementary material for: Polygenic Risk Score Modifies Prostate Cancer Risk of Pathogenic Variants in Men of African Ancestry
Source: Cancer Res Commun. 2023 Dec 14;3(12):2544–50. doi: 10.1158/2767-9764.CRC-23-0022 (PMC10720390; doi:10.1158/2767-9764.CRC-23-0022)
Supplement: Supplementary Table 14 — Association of PRS and PCa risk in Ugandan men. [file crc-23-0022-s15.docx]

**Supplementary Table 14.** Association of PRS and PCa risk in Ugandan men.

|  | **PRS Category** | **N Controls** | **N Cases** | **OR** | **95% CI** | **P value** |
| --- | --- | --- | --- | --- | --- | --- |
| **Overall PCa**  **versus controls** | Low PRS | 120 | 41 | 0.43 | 0.27 to 0.68 | 2.00 x10^−4^ |
|  | Intermediate PRS | 143 | 116 | Ref | -- | -- |
|  | High PRS | 196 | 353 | 2.41 | 1.74 to 3.34 | 1.26 x10^-07^ |
| **Metastatic PCa**  **versus controls** | Low PRS | 120 | 12 | 0.46 | 0.22 to 0.96 | 0.040 |
|  | Intermediate PRS | 143 | 30 | Ref | -- | -- |
|  | High PRS | 196 | 110 | 2.82 | 1.70 to 4.66 | 5.71x10^-05^ |
| **Aggressive PCa**  **versus controls** | Low PRS | 120 | 22 | 0.37 | 0.21 to 0.66 | 0.001 |
|  | Intermediate PRS | 143 | 68 | Ref | -- | -- |
|  | High PRS | 196 | 222 | 2.56 | 1.75 to 3.73 | 1.16x10^-06^ |
| **Non-aggressive PCa**  **versus controls** | Low PRS | 120 | 5 | 0.61 | 0.20 to 1.90 | 0.395 |
|  | Intermediate PRS | 143 | 10 | Ref | -- | -- |
|  | High PRS | 196 | 25 | 2.17 | 0.96 to 4.89 | 0.063 |
