# Supplementary material for: Polygenic Risk Score Modifies Prostate Cancer Risk of Pathogenic Variants in Men of African Ancestry
Source: Cancer Res Commun. 2023 Dec 14;3(12):2544–50. doi: 10.1158/2767-9764.CRC-23-0022 (PMC10720390; doi:10.1158/2767-9764.CRC-23-0022)
Supplement: Supplementary Table 15 — Aggregate effect of PRS and P/LP/D variants in BRCA2, ATM, NBN, and PALB2 on PCa risk in Ugandan men. [file crc-23-0022-s16.docx]

**Supplementary Table 15.** Aggregate effect of PRS and P/LP/D variants in *BRCA2*, *ATM*, *NBN*, and *PALB2* on PCa risk in Ugandan men.

|  | **PRS Category** | **Carrier Status** | **N Controls** | **N Cases** | **OR** | **95% CI** | **P value** |
| --- | --- | --- | --- | --- | --- | --- | --- |
| **Overall PCa**  **versus controls** | Low PRS | Non-Carrier | 119 | 39 | 0.43 | 0.27 to 0.68 | 3.00 x10^−4^ |
|  | Low PRS | Carrier | 1 | 2 | 3.51 | 0.26 to 47.37 | 0.345 |
|  | Intermediate PRS | Non-Carrier | 143 | 111 | Ref | -- | -- |
|  | Intermediate PRS | Carrier | 0 | 5 | NA | NA | NA |
|  | High PRS | Non-Carrier | 195 | 338 | 2.41 | 1.73 to 3.35 | 1.79x10^-07^ |
|  | High PRS | Carrier | 1 | 15 | 22.70 | 2.81 to 183.11 | 0.003 |
| **Metastatic PCa**  **versus controls** | Low PRS | Non-Carrier | 119 | 11 | 0.47 | 0.22 to 1.03 | 0.059 |
|  | Low PRS | Carrier | 1 | 1 | 3.82 | 0.16 to 93.27 | 0.411 |
|  | Intermediate PRS | Non-Carrier | 143 | 27 | Ref | -- | -- |
|  | Intermediate PRS | Carrier | 0 | 3 | NA | NA | NA |
|  | High PRS | Non-Carrier | 195 | 106 | 3.01 | 1.79 to 5.08 | 3.34x10^-05^ |
|  | High PRS | Carrier | 1 | 4 | 24.00 | 2.23 to 258.52 | 0.009 |
| **Aggressive PCa**  **versus controls** | Low PRS | Non-Carrier | 119 | 21 | 0.38 | 0.22 to 0.68 | 0.001 |
|  | Low PRS | Carrier | 1 | 1 | 1.59 | 0.08 to 31.53 | 0.76 |
|  | Intermediate PRS | Non-Carrier | 143 | 64 | Ref | -- | -- |
|  | Intermediate PRS | Carrier | 0 | 4 | NA | NA | NA |
|  | High PRS | Non-Carrier | 195 | 211 | 2.58 | 1.76 to 3.79 | 1.30x10^-06^ |
|  | High PRS | Carrier | 1 | 11 | 24.07 | 2.86 to 202.32 | 0.003 |
| **Non-aggressive PCa versus controls** | Low PRS | Non-Carrier | 119 | 5 | 0.61 | 0.20 to 1.90 | 0.398 |
|  | Low PRS | Carrier | 1 | 0 | NA | NA | NA |
|  | Intermediate PRS | Non-Carrier | 143 | 10 | Ref | -- | -- |
|  | Intermediate PRS | Carrier | 0 | 0 | NA | NA | NA |
|  | High PRS | Non-Carrier | 195 | 25 | 2.17 | 0.96 to 4.89 | 0.063 |
|  | High PRS | Carrier | 1 | 0 | NA | NA | NA |
