# Supplementary material for: Polygenic Risk Score Modifies Prostate Cancer Risk of Pathogenic Variants in Men of African Ancestry
Source: Cancer Res Commun. 2023 Dec 14;3(12):2544–50. doi: 10.1158/2767-9764.CRC-23-0022 (PMC10720390; doi:10.1158/2767-9764.CRC-23-0022)
Supplement: Supplementary Table 16 — Aggregate effect of P/LP/D carrier status across BRCA1, RAD50, MLH1, and MSH6 genes on PCa risk in African ancestry men. [file crc-23-0022-s17.docx]

**Supplementary Table 16.** Aggregate effect of P/LP/D carrier status across *BRCA1, RAD50, MLH1*, and *MSH6* genes on PCa risk in African ancestry men.

|  | **Carrier Status** | **N Controls** | **N Cases** | **OR** | **95% CI** | **P value** |
| --- | --- | --- | --- | --- | --- | --- |
| **Overall PCa**  **versus controls** | Non-Carrier | 1417 | 1781 | Ref | -- | -- |
|  | Carrier | 7 | 15 | 1.64 | 0.66 to 4.07 | 0.286 |
| **Metastatic PCa**  **versus controls** | Non-Carrier | 1417 | 220 | Ref | -- | -- |
|  | Carrier | 7 | 2 | 1.08 | 0.21 to 5.64 | 0.926 |
| **Aggressive PCa**  **versus controls** | Non-Carrier | 1417 | 892 | Ref | -- | -- |
|  | Carrier | 7 | 11 | 2.4 | 0.92 to 6.25 | 0.074 |
| **Non-aggressive PCa**  **versus controls** | Non-Carrier | 1417 | 731 | Ref | -- | -- |
|  | Carrier | 7 | 4 | 1.36 | 0.32 to 5.84 | 0.680 |
