# Supplementary material for: Polygenic Risk Score Modifies Prostate Cancer Risk of Pathogenic Variants in Men of African Ancestry
Source: Cancer Res Commun. 2023 Dec 14;3(12):2544–50. doi: 10.1158/2767-9764.CRC-23-0022 (PMC10720390; doi:10.1158/2767-9764.CRC-23-0022)
Supplement: Supplementary Table 17 — Aggregate effect of PRS and P/LP/D variants in BRCA1, RAD50, MLH1, and MSH6 on PCa risk in African ancestry men. [file crc-23-0022-s18.docx]

**Supplementary Table 17.** Aggregate effect of PRS and P/LP/D variants in *BRCA1, RAD50, MLH1*, and *MSH6* on PCa risk in African ancestry men.

|  | **PRS Category** | **Carrier Status** | **N Controls** | **N Cases** | **OR** | **95% CI** | **P value** |
| --- | --- | --- | --- | --- | --- | --- | --- |
| **Overall PCa**  **versus controls** | Low PRS | Non-Carrier | 475 | 223 | 0.56 | 0.45 to 0.69 | 6.77x10^-08^ |
|  | Low PRS | Carrier | 0 | 5 | NA | NA | NA |
|  | Intermediate PRS | Non-Carrier | 470 | 387 | Ref | -- | -- |
|  | Intermediate PRS | Carrier | 4 | 4 | 1.47 | 0.36 to 6.00 | 0.595 |
|  | High PRS | Non-Carrier | 472 | 1171 | 3.02 | 2.54 to 3.60 | 1.89x10^-35^ |
|  | High PRS | Carrier | 3 | 6 | 2.14 | 0.53 to 8.69 | 0.289 |
| **Metastatic PCa**  **versus controls** | Low PRS | Non-Carrier | 475 | 21 | 0.53 | 0.31 to 0.92 | 0.024 |
|  | Low PRS | Carrier | 0 | 0 | NA | NA | NA |
|  | Intermediate PRS | Non-Carrier | 470 | 43 | Ref | -- | -- |
|  | Intermediate PRS | Carrier | 4 | 1 | 1.72 | 0.16 to 18.05 | 0.650 |
|  | High PRS | Non-Carrier | 472 | 156 | 3.15 | 2.16 to 4.60 | 2.61x10^-09^ |
|  | High PRS | Carrier | 3 | 1 | 2.02 | 0.19 to 21.11 | 0.556 |
| **Aggressive PCa**  **versus controls** | Low PRS | Non-Carrier | 475 | 99 | 0.52 | 0.40 to 0.69 | 3.74x10^-06^ |
|  | Low PRS | Carrier | 0 | 2 | NA | NA | NA |
|  | Intermediate PRS | Non-Carrier | 470 | 188 | Ref | -- | -- |
|  | Intermediate PRS | Carrier | 4 | 4 | 2.76 | 0.67 to 11.33 | 0.159 |
|  | High PRS | Non-Carrier | 472 | 605 | 3.18 | 2.57 to 3.92 | 4.99x10^-27^ |
|  | High PRS | Carrier | 3 | 5 | 3.68 | 0.87 to 15.64 | 0.077 |
| **Non-aggressive PCa versus controls** | Low PRS | Non-Carrier | 475 | 110 | 0.68 | 0.51 to 0.91 | 0.008 |
|  | Low PRS | Carrier | 0 | 3 | NA | NA | NA |
|  | Intermediate PRS | Non-Carrier | 470 | 161 | Ref | -- | -- |
|  | Intermediate PRS | Carrier | 4 | 0 | NA | NA | NA |
|  | High PRS | Non-Carrier | 472 | 460 | 3.09 | 2.45 to 3.90 | 2.20x10^-21^ |
|  | High PRS | Carrier | 3 | 1 | 0.82 | 0.08 to 8.71 | 0.873 |
