# Supplementary material for: Polygenic Risk Score Modifies Prostate Cancer Risk of Pathogenic Variants in Men of African Ancestry
Source: Cancer Res Commun. 2023 Dec 14;3(12):2544–50. doi: 10.1158/2767-9764.CRC-23-0022 (PMC10720390; doi:10.1158/2767-9764.CRC-23-0022)
Supplement: Supplementary Table 18 — Absolute risk of PCa by PRS and P/LP/D variants in BRCA2, ATM, NBN, and PALB2 independently in African ancestry men. [file crc-23-0022-s19.docx]

**Supplementary Table 18.** Absolute risk of PCa by PRS and P/LP/D variants in *BRCA2*, *ATM*, *NBN*, and *PALB2* independently in African ancestry men. Absolute risks are based on the weighted PCa OR reported in Supplementary Table S2 to account for the over-representation of aggressive cases in this sample.

|  | **Absolute Risk(%) and 95% CI** | | **Absolute Risk (%) and 95% CI** | | |
| --- | --- | --- | --- | --- | --- |
|  | **by Carrier Status** | | **by PRS Category** | | |
| **Age** | Carrier | Non-Carrier | Low PRS | Intermediate PRS | High PRS |
| 40 | 0.01 (0 to 0.03) | 0 (0 to 0) | 0 (0 to 0) | 0 (0 to 0) | 0.01 (0.01 to 0.01) |
| 41 | 0.05 (0 to 0.12) | 0.02 (0.02 to 0.02) | 0.01 (0.01 to 0.01) | 0.01 (0.01 to 0.02) | 0.04 (0.04 to 0.05) |
| 42 | 0.1 (0.01 to 0.21) | 0.04 (0.04 to 0.04) | 0.01 (0.01 to 0.02) | 0.02 (0.02 to 0.03) | 0.07 (0.07 to 0.08) |
| 43 | 0.14 (0.01 to 0.3) | 0.05 (0.05 to 0.06) | 0.02 (0.01 to 0.03) | 0.03 (0.03 to 0.04) | 0.11 (0.1 to 0.12) |
| 44 | 0.18 (0.01 to 0.39) | 0.07 (0.07 to 0.07) | 0.03 (0.02 to 0.04) | 0.04 (0.04 to 0.05) | 0.14 (0.13 to 0.15) |
| 45 | 0.22 (0.01 to 0.48) | 0.09 (0.08 to 0.09) | 0.03 (0.02 to 0.04) | 0.05 (0.05 to 0.06) | 0.17 (0.15 to 0.19) |
| 46 | 0.41 (0.03 to 0.9) | 0.16 (0.16 to 0.17) | 0.06 (0.04 to 0.08) | 0.1 (0.09 to 0.12) | 0.32 (0.29 to 0.35) |
| 47 | 0.61 (0.04 to 1.31) | 0.24 (0.23 to 0.24) | 0.09 (0.07 to 0.12) | 0.15 (0.13 to 0.18) | 0.47 (0.42 to 0.51) |
| 48 | 0.8 (0.05 to 1.72) | 0.31 (0.3 to 0.32) | 0.12 (0.09 to 0.16) | 0.2 (0.17 to 0.23) | 0.61 (0.56 to 0.67) |
| 49 | 0.99 (0.06 to 2.13) | 0.38 (0.37 to 0.39) | 0.15 (0.11 to 0.2) | 0.25 (0.21 to 0.29) | 0.76 (0.69 to 0.83) |
| 50 | 1.17 (0.08 to 2.53) | 0.46 (0.44 to 0.47) | 0.18 (0.13 to 0.24) | 0.29 (0.25 to 0.34) | 0.91 (0.82 to 0.98) |
| 51 | 1.72 (0.12 to 3.69) | 0.67 (0.65 to 0.69) | 0.27 (0.19 to 0.35) | 0.43 (0.36 to 0.5) | 1.33 (1.2 to 1.44) |
| 52 | 2.26 (0.16 to 4.84) | 0.88 (0.86 to 0.9) | 0.35 (0.25 to 0.46) | 0.56 (0.48 to 0.66) | 1.74 (1.58 to 1.89) |
| 53 | 2.78 (0.21 to 5.95) | 1.09 (1.06 to 1.12) | 0.43 (0.31 to 0.57) | 0.7 (0.59 to 0.81) | 2.16 (1.95 to 2.34) |
| 54 | 3.31 (0.27 to 7.05) | 1.3 (1.26 to 1.33) | 0.52 (0.36 to 0.68) | 0.83 (0.71 to 0.97) | 2.56 (2.32 to 2.78) |
| 55 | 3.82 (0.32 to 8.12) | 1.5 (1.46 to 1.54) | 0.6 (0.42 to 0.79) | 0.96 (0.82 to 1.12) | 2.96 (2.68 to 3.22) |
| 56 | 4.8 (0.44 to 10.15) | 1.89 (1.84 to 1.93) | 0.76 (0.53 to 1) | 1.21 (1.03 to 1.41) | 3.73 (3.38 to 4.05) |
| 57 | 5.75 (0.57 to 12.1) | 2.27 (2.21 to 2.32) | 0.91 (0.64 to 1.2) | 1.46 (1.24 to 1.7) | 4.48 (4.05 to 4.86) |
| 58 | 6.67 (0.71 to 13.98) | 2.65 (2.57 to 2.71) | 1.06 (0.75 to 1.4) | 1.7 (1.45 to 1.99) | 5.21 (4.72 to 5.65) |
| 59 | 7.58 (0.86 to 15.79) | 3.02 (2.93 to 3.08) | 1.21 (0.86 to 1.6) | 1.95 (1.66 to 2.26) | 5.93 (5.37 to 6.43) |
| 60 | 8.46 (1.02 to 17.53) | 3.38 (3.28 to 3.45) | 1.36 (0.96 to 1.79) | 2.18 (1.86 to 2.54) | 6.63 (6.01 to 7.19) |
| 61 | 9.81 (1.28 to 20.17) | 3.94 (3.83 to 4.02) | 1.59 (1.12 to 2.09) | 2.55 (2.17 to 2.96) | 7.71 (6.99 to 8.37) |
| 62 | 11.11 (1.57 to 22.66) | 4.48 (4.36 to 4.57) | 1.82 (1.29 to 2.38) | 2.91 (2.48 to 3.38) | 8.76 (7.93 to 9.5) |
| 63 | 12.35 (1.87 to 25) | 5 (4.87 to 5.11) | 2.04 (1.44 to 2.67) | 3.26 (2.78 to 3.78) | 9.77 (8.85 to 10.6) |
| 64 | 13.55 (2.19 to 27.22) | 5.52 (5.38 to 5.63) | 2.25 (1.6 to 2.95) | 3.6 (3.07 to 4.18) | 10.75 (9.74 to 11.66) |
| 65 | 14.71 (2.52 to 29.31) | 6.02 (5.86 to 6.14) | 2.46 (1.75 to 3.23) | 3.93 (3.36 to 4.56) | 11.7 (10.61 to 12.69) |
| 66 | 16.27 (3.01 to 32.1) | 6.7 (6.54 to 6.84) | 2.76 (1.96 to 3.61) | 4.4 (3.76 to 5.1) | 13 (11.79 to 14.1) |
| 67 | 17.76 (3.51 to 34.67) | 7.36 (7.18 to 7.51) | 3.04 (2.17 to 3.98) | 4.84 (4.15 to 5.61) | 14.24 (12.91 to 15.44) |
| 68 | 19.16 (4.03 to 37.05) | 7.99 (7.81 to 8.15) | 3.32 (2.37 to 4.33) | 5.28 (4.53 to 6.11) | 15.43 (13.99 to 16.73) |
| 69 | 20.49 (4.56 to 39.23) | 8.6 (8.41 to 8.77) | 3.59 (2.56 to 4.68) | 5.7 (4.9 to 6.59) | 16.56 (15.03 to 17.96) |
| 70 | 21.75 (5.1 to 41.25) | 9.19 (8.99 to 9.36) | 3.85 (2.76 to 5.01) | 6.11 (5.25 to 7.06) | 17.65 (16.01 to 19.14) |
| 71 | 22.81 (5.58 to 42.91) | 9.69 (9.48 to 9.87) | 4.08 (2.92 to 5.31) | 6.46 (5.56 to 7.46) | 18.58 (16.86 to 20.14) |
| 72 | 23.81 (6.06 to 44.43) | 10.17 (9.96 to 10.36) | 4.3 (3.08 to 5.58) | 6.81 (5.86 to 7.85) | 19.45 (17.65 to 21.09) |
| 73 | 24.75 (6.54 to 45.83) | 10.63 (10.41 to 10.82) | 4.51 (3.24 to 5.85) | 7.13 (6.15 to 8.22) | 20.29 (18.41 to 21.99) |
| 74 | 25.63 (7 to 47.11) | 11.07 (10.84 to 11.26) | 4.71 (3.39 to 6.11) | 7.45 (6.42 to 8.57) | 21.08 (19.13 to 22.84) |
| 75 | 26.46 (7.46 to 48.3) | 11.48 (11.25 to 11.68) | 4.9 (3.54 to 6.36) | 7.75 (6.69 to 8.91) | 21.83 (19.82 to 23.66) |
| 76 | 27.11 (7.83 to 49.19) | 11.81 (11.58 to 12.01) | 5.06 (3.65 to 6.56) | 7.99 (6.9 to 9.18) | 22.42 (20.36 to 24.29) |
| 77 | 27.71 (8.18 to 50.01) | 12.12 (11.89 to 12.32) | 5.21 (3.76 to 6.74) | 8.22 (7.11 to 9.44) | 22.97 (20.86 to 24.89) |
| 78 | 28.27 (8.52 to 50.76) | 12.41 (12.18 to 12.62) | 5.35 (3.87 to 6.92) | 8.43 (7.3 to 9.68) | 23.49 (21.33 to 25.45) |
| 79 | 28.79 (8.85 to 51.44) | 12.69 (12.45 to 12.89) | 5.48 (3.97 to 7.09) | 8.64 (7.48 to 9.91) | 23.97 (21.77 to 25.97) |
| 80 | 29.27 (9.16 to 52.06) | 12.94 (12.71 to 13.15) | 5.61 (4.07 to 7.24) | 8.83 (7.65 to 10.13) | 24.42 (22.18 to 26.46) |
| 81 | 29.58 (9.36 to 52.45) | 13.11 (12.87 to 13.32) | 5.69 (4.13 to 7.35) | 8.96 (7.77 to 10.27) | 24.71 (22.45 to 26.77) |
| 82 | 29.86 (9.55 to 52.8) | 13.27 (13.03 to 13.48) | 5.77 (4.19 to 7.44) | 9.07 (7.87 to 10.4) | 24.98 (22.7 to 27.06) |
| 83 | 30.12 (9.73 to 53.11) | 13.41 (13.17 to 13.62) | 5.84 (4.24 to 7.53) | 9.18 (7.97 to 10.52) | 25.23 (22.92 to 27.33) |
| 84 | 30.35 (9.89 to 53.4) | 13.54 (13.3 to 13.75) | 5.91 (4.29 to 7.61) | 9.28 (8.06 to 10.63) | 25.45 (23.13 to 27.57) |
| 85 | 30.57 (10.04 to 53.65) | 13.66 (13.42 to 13.87) | 5.97 (4.34 to 7.69) | 9.37 (8.14 to 10.73) | 25.66 (23.32 to 27.79) |
