# Supplementary material for: Polygenic Risk Score Modifies Prostate Cancer Risk of Pathogenic Variants in Men of African Ancestry
Source: Cancer Res Commun. 2023 Dec 14;3(12):2544–50. doi: 10.1158/2767-9764.CRC-23-0022 (PMC10720390; doi:10.1158/2767-9764.CRC-23-0022)
Supplement: Supplementary Table 19 — Absolute risk of metastatic PCa by PRS and P/LP/D variants in BRCA2, ATM, NBN, and PALB2 independently in African ancestry men. [file crc-23-0022-s20.docx]

**Supplementary Table 19.** Absolute risk of metastatic PCa by PRS and P/LP/D variants in *BRCA2*, *ATM*, *NBN*, and *PALB2* independently in African ancestry men.

|  | **Absolute Risk(%) and 95% CI** | | **Absolute Risk (%) and 95% CI** | | |
| --- | --- | --- | --- | --- | --- |
|  | **by Carrier Status** | | **by PRS Category** | | |
| Age | Carrier | Non-Carrier | Low PRS | Intermediate PRS | High PRS |
| 40 | 0 (0 to 0) | 0 (0 to 0) | 0 (0 to 0) | 0 (0 to 0) | 0 (0 to 0) |
| 41 | 0 (0 to 0) | 0 (0 to 0) | 0 (0 to 0) | 0 (0 to 0) | 0 (0 to 0) |
| 42 | 0 (0 to 0) | 0 (0 to 0) | 0 (0 to 0) | 0 (0 to 0) | 0 (0 to 0) |
| 43 | 0 (0 to 0) | 0 (0 to 0) | 0 (0 to 0) | 0 (0 to 0) | 0 (0 to 0) |
| 44 | 0 (0 to 0) | 0 (0 to 0) | 0 (0 to 0) | 0 (0 to 0) | 0 (0 to 0) |
| 45 | 0 (0 to 0) | 0 (0 to 0) | 0 (0 to 0) | 0 (0 to 0) | 0 (0 to 0) |
| 46 | 0.03 (0 to 0.06) | 0 (0 to 0) | 0 (0 to 0) | 0 (0 to 0) | 0.01 (0.01 to 0.01) |
| 47 | 0.05 (0 to 0.13) | 0.01 (0.01 to 0.01) | 0 (0 to 0) | 0.01 (0 to 0.01) | 0.02 (0.01 to 0.02) |
| 48 | 0.08 (0 to 0.19) | 0.01 (0.01 to 0.01) | 0 (0 to 0.01) | 0.01 (0.01 to 0.01) | 0.02 (0.02 to 0.03) |
| 49 | 0.11 (0 to 0.25) | 0.02 (0.01 to 0.02) | 0.01 (0 to 0.01) | 0.01 (0.01 to 0.01) | 0.03 (0.03 to 0.04) |
| 50 | 0.13 (0 to 0.32) | 0.02 (0.02 to 0.02) | 0.01 (0 to 0.01) | 0.01 (0.01 to 0.02) | 0.04 (0.04 to 0.05) |
| 51 | 0.21 (0 to 0.49) | 0.03 (0.03 to 0.03) | 0.01 (0 to 0.02) | 0.02 (0.02 to 0.03) | 0.06 (0.05 to 0.07) |
| 52 | 0.28 (0 to 0.66) | 0.04 (0.04 to 0.04) | 0.01 (0.01 to 0.02) | 0.03 (0.02 to 0.03) | 0.09 (0.07 to 0.1) |
| 53 | 0.35 (0 to 0.83) | 0.05 (0.05 to 0.05) | 0.02 (0.01 to 0.03) | 0.03 (0.03 to 0.04) | 0.11 (0.09 to 0.12) |
| 54 | 0.42 (0 to 0.99) | 0.06 (0.06 to 0.07) | 0.02 (0.01 to 0.04) | 0.04 (0.03 to 0.05) | 0.13 (0.11 to 0.15) |
| 55 | 0.49 (0 to 1.16) | 0.07 (0.06 to 0.08) | 0.03 (0.01 to 0.04) | 0.05 (0.04 to 0.06) | 0.15 (0.13 to 0.17) |
| 56 | 0.64 (0 to 1.51) | 0.09 (0.08 to 0.1) | 0.03 (0.02 to 0.06) | 0.06 (0.05 to 0.08) | 0.2 (0.17 to 0.23) |
| 57 | 0.79 (0 to 1.86) | 0.11 (0.1 to 0.12) | 0.04 (0.02 to 0.07) | 0.08 (0.06 to 0.1) | 0.24 (0.21 to 0.28) |
| 58 | 0.94 (0 to 2.2) | 0.14 (0.12 to 0.15) | 0.05 (0.02 to 0.08) | 0.09 (0.07 to 0.11) | 0.29 (0.25 to 0.33) |
| 59 | 1.08 (0 to 2.54) | 0.16 (0.14 to 0.17) | 0.06 (0.03 to 0.1) | 0.11 (0.08 to 0.13) | 0.33 (0.28 to 0.38) |
| 60 | 1.22 (0 to 2.87) | 0.18 (0.16 to 0.19) | 0.06 (0.03 to 0.11) | 0.12 (0.09 to 0.15) | 0.38 (0.32 to 0.43) |
| 61 | 1.51 (0 to 3.54) | 0.22 (0.2 to 0.24) | 0.08 (0.04 to 0.13) | 0.15 (0.11 to 0.18) | 0.47 (0.4 to 0.54) |
| 62 | 1.79 (0 to 4.18) | 0.26 (0.24 to 0.28) | 0.09 (0.04 to 0.16) | 0.18 (0.13 to 0.22) | 0.56 (0.47 to 0.64) |
| 63 | 2.07 (0 to 4.81) | 0.3 (0.27 to 0.32) | 0.11 (0.05 to 0.18) | 0.21 (0.15 to 0.25) | 0.64 (0.54 to 0.74) |
| 64 | 2.33 (0 to 5.42) | 0.34 (0.31 to 0.37) | 0.12 (0.06 to 0.21) | 0.23 (0.17 to 0.28) | 0.72 (0.61 to 0.83) |
| 65 | 2.59 (0 to 6.02) | 0.38 (0.34 to 0.41) | 0.14 (0.06 to 0.23) | 0.26 (0.19 to 0.32) | 0.81 (0.68 to 0.93) |
| 66 | 2.91 (0 to 6.74) | 0.43 (0.39 to 0.46) | 0.16 (0.07 to 0.26) | 0.29 (0.21 to 0.36) | 0.91 (0.77 to 1.04) |
| 67 | 3.22 (0 to 7.43) | 0.47 (0.43 to 0.51) | 0.17 (0.08 to 0.29) | 0.32 (0.24 to 0.39) | 1 (0.85 to 1.15) |
| 68 | 3.51 (0 to 8.09) | 0.52 (0.47 to 0.55) | 0.19 (0.09 to 0.31) | 0.35 (0.26 to 0.43) | 1.1 (0.93 to 1.26) |
| 69 | 3.8 (0 to 8.73) | 0.56 (0.51 to 0.6) | 0.2 (0.09 to 0.34) | 0.38 (0.28 to 0.47) | 1.19 (1.01 to 1.36) |
| 70 | 4.08 (0 to 9.34) | 0.6 (0.55 to 0.64) | 0.22 (0.1 to 0.37) | 0.41 (0.3 to 0.5) | 1.28 (1.08 to 1.47) |
| 71 | 4.42 (0 to 10.1) | 0.65 (0.6 to 0.7) | 0.24 (0.11 to 0.4) | 0.45 (0.33 to 0.55) | 1.39 (1.18 to 1.59) |
| 72 | 4.75 (0 to 10.82) | 0.7 (0.64 to 0.75) | 0.26 (0.12 to 0.43) | 0.48 (0.35 to 0.59) | 1.49 (1.27 to 1.71) |
| 73 | 5.07 (0 to 11.5) | 0.75 (0.69 to 0.8) | 0.27 (0.12 to 0.46) | 0.52 (0.38 to 0.63) | 1.6 (1.35 to 1.83) |
| 74 | 5.37 (0 to 12.15) | 0.8 (0.73 to 0.85) | 0.29 (0.13 to 0.49) | 0.55 (0.4 to 0.67) | 1.69 (1.43 to 1.94) |
| 75 | 5.66 (0 to 12.76) | 0.84 (0.77 to 0.9) | 0.31 (0.14 to 0.51) | 0.58 (0.42 to 0.71) | 1.79 (1.51 to 2.05) |
| 76 | 5.97 (0 to 13.43) | 0.89 (0.82 to 0.95) | 0.33 (0.15 to 0.54) | 0.61 (0.45 to 0.75) | 1.89 (1.6 to 2.17) |
| 77 | 6.27 (0.03 to 14.05) | 0.94 (0.86 to 1) | 0.34 (0.16 to 0.57) | 0.64 (0.47 to 0.79) | 1.99 (1.68 to 2.28) |
| 78 | 6.55 (0.06 to 14.63) | 0.98 (0.9 to 1.05) | 0.36 (0.16 to 0.6) | 0.67 (0.5 to 0.82) | 2.08 (1.76 to 2.39) |
| 79 | 6.81 (0.1 to 15.17) | 1.02 (0.94 to 1.09) | 0.37 (0.17 to 0.62) | 0.7 (0.52 to 0.86) | 2.17 (1.84 to 2.49) |
| 80 | 7.06 (0.13 to 15.67) | 1.06 (0.97 to 1.13) | 0.39 (0.18 to 0.65) | 0.73 (0.54 to 0.89) | 2.25 (1.91 to 2.58) |
| 81 | 7.31 (0.18 to 16.19) | 1.1 (1.01 to 1.18) | 0.4 (0.18 to 0.67) | 0.76 (0.56 to 0.92) | 2.33 (1.98 to 2.68) |
| 82 | 7.55 (0.22 to 16.66) | 1.14 (1.05 to 1.22) | 0.42 (0.19 to 0.7) | 0.78 (0.58 to 0.96) | 2.41 (2.05 to 2.77) |
| 83 | 7.76 (0.26 to 17.08) | 1.18 (1.08 to 1.25) | 0.43 (0.2 to 0.72) | 0.81 (0.59 to 0.98) | 2.49 (2.11 to 2.85) |
| 84 | 7.96 (0.3 to 17.47) | 1.21 (1.11 to 1.29) | 0.44 (0.2 to 0.74) | 0.83 (0.61 to 1.01) | 2.55 (2.16 to 2.93) |
| 85 | 8.15 (0.34 to 17.82) | 1.24 (1.14 to 1.32) | 0.45 (0.21 to 0.76) | 0.85 (0.63 to 1.04) | 2.62 (2.22 to 3) |
