# Supplementary material for: Polygenic Risk Score Modifies Prostate Cancer Risk of Pathogenic Variants in Men of African Ancestry
Source: Cancer Res Commun. 2023 Dec 14;3(12):2544–50. doi: 10.1158/2767-9764.CRC-23-0022 (PMC10720390; doi:10.1158/2767-9764.CRC-23-0022)
Supplement: Supplementary Table 20 — Absolute risk of PCa by PRS and P/LP/D variants in BRCA2, ATM, NBN, and PALB2 combined in African ancestry men. [file crc-23-0022-s21.docx]

**Supplementary Table 20.** Absolute risk of PCa by PRS and P/LP/D variants in *BRCA2*, *ATM*, *NBN*, and *PALB2* combined in African ancestry men. Absolute risks reported here are based on the weighted PCa OR reported in Supplementary Table 3 to account for the over-representation of aggressive cases in this sample.

| **Absolute Risk(%) and 95% CI by Combined PRS and Carrier Status Categories** | | | | | | |
| --- | --- | --- | --- | --- | --- | --- |
| **Age** | **Low PRS Non−Carrier** | **Low PRS Carrier** | **Intermediate PRS Non−Carrier** | **Intermediate PRS Carrier** | **High PRS Non−Carrier** | **High PRS Carrier** |
| 40 | 0 (0 to 0) | 0 (0 to 0.01) | 0 (0 to 0) | 0.01 (0 to 0.04) | 0.01 (0.01 to 0.01) | 0.03 (0 to 0.07) |
| 41 | 0.01 (0.01 to 0.01) | 0.01 (0 to 0.06) | 0.01 (0.01 to 0.01) | 0.05 (0 to 0.18) | 0.04 (0.04 to 0.04) | 0.11 (0 to 0.32) |
| 42 | 0.01 (0.01 to 0.02) | 0.03 (0 to 0.11) | 0.02 (0.02 to 0.03) | 0.08 (0 to 0.31) | 0.07 (0.06 to 0.07) | 0.2 (0 to 0.56) |
| 43 | 0.02 (0.01 to 0.03) | 0.04 (0 to 0.15) | 0.03 (0.03 to 0.04) | 0.12 (0 to 0.44) | 0.1 (0.09 to 0.11) | 0.28 (0 to 0.8) |
| 44 | 0.03 (0.02 to 0.03) | 0.05 (0 to 0.2) | 0.04 (0.03 to 0.05) | 0.15 (0 to 0.58) | 0.13 (0.12 to 0.14) | 0.37 (0 to 1.04) |
| 45 | 0.03 (0.02 to 0.04) | 0.06 (0 to 0.25) | 0.05 (0.04 to 0.06) | 0.19 (0 to 0.71) | 0.16 (0.14 to 0.17) | 0.45 (0 to 1.28) |
| 46 | 0.06 (0.04 to 0.08) | 0.11 (0 to 0.46) | 0.09 (0.08 to 0.11) | 0.35 (0 to 1.33) | 0.29 (0.27 to 0.32) | 0.85 (0 to 2.39) |
| 47 | 0.09 (0.06 to 0.12) | 0.16 (0 to 0.68) | 0.14 (0.11 to 0.16) | 0.52 (0 to 1.94) | 0.43 (0.39 to 0.46) | 1.24 (0 to 3.48) |
| 48 | 0.11 (0.08 to 0.15) | 0.21 (0 to 0.89) | 0.18 (0.15 to 0.21) | 0.68 (0 to 2.55) | 0.57 (0.52 to 0.61) | 1.62 (0 to 4.56) |
| 49 | 0.14 (0.1 to 0.19) | 0.26 (0 to 1.1) | 0.22 (0.18 to 0.26) | 0.84 (0 to 3.14) | 0.7 (0.64 to 0.75) | 2.01 (0 to 5.61) |
| 50 | 0.17 (0.12 to 0.22) | 0.31 (0 to 1.31) | 0.27 (0.22 to 0.31) | 1 (0 to 3.73) | 0.83 (0.76 to 0.89) | 2.39 (0 to 6.65) |
| 51 | 0.25 (0.18 to 0.33) | 0.45 (0 to 1.92) | 0.39 (0.32 to 0.45) | 1.46 (0 to 5.43) | 1.22 (1.12 to 1.31) | 3.49 (0 to 9.65) |
| 52 | 0.32 (0.24 to 0.43) | 0.59 (0 to 2.51) | 0.51 (0.42 to 0.59) | 1.92 (0 to 7.08) | 1.61 (1.47 to 1.72) | 4.57 (0 to 12.52) |
| 53 | 0.4 (0.29 to 0.53) | 0.73 (0 to 3.1) | 0.64 (0.52 to 0.73) | 2.37 (0 to 8.69) | 1.99 (1.81 to 2.13) | 5.63 (0 to 15.28) |
| 54 | 0.48 (0.35 to 0.64) | 0.87 (0 to 3.68) | 0.76 (0.62 to 0.87) | 2.82 (0 to 10.24) | 2.36 (2.15 to 2.53) | 6.66 (0 to 17.93) |
| 55 | 0.56 (0.4 to 0.74) | 1.01 (0 to 4.25) | 0.88 (0.72 to 1.01) | 3.26 (0 to 11.76) | 2.73 (2.49 to 2.93) | 7.68 (0 to 20.48) |
| 56 | 0.7 (0.51 to 0.93) | 1.27 (0 to 5.33) | 1.11 (0.91 to 1.28) | 4.1 (0 to 14.59) | 3.44 (3.14 to 3.68) | 9.6 (0 to 25.19) |
| 57 | 0.84 (0.62 to 1.12) | 1.53 (0 to 6.38) | 1.33 (1.1 to 1.54) | 4.92 (0 to 17.28) | 4.13 (3.77 to 4.42) | 11.45 (0 to 29.56) |
| 58 | 0.99 (0.72 to 1.31) | 1.79 (0 to 7.41) | 1.56 (1.28 to 1.79) | 5.72 (0 to 19.83) | 4.81 (4.39 to 5.15) | 13.24 (0 to 33.62) |
| 59 | 1.12 (0.82 to 1.49) | 2.04 (0 to 8.4) | 1.78 (1.46 to 2.04) | 6.51 (0 to 22.25) | 5.47 (5 to 5.86) | 14.97 (0 to 37.4) |
| 60 | 1.26 (0.92 to 1.68) | 2.29 (0 to 9.37) | 1.99 (1.64 to 2.29) | 7.28 (0 to 24.56) | 6.12 (5.6 to 6.55) | 16.63 (0 to 40.91) |
| 61 | 1.48 (1.08 to 1.96) | 2.67 (0 to 10.85) | 2.33 (1.92 to 2.68) | 8.46 (0 to 28) | 7.12 (6.52 to 7.63) | 19.15 (0 to 46.03) |
| 62 | 1.68 (1.24 to 2.23) | 3.05 (0 to 12.27) | 2.65 (2.19 to 3.05) | 9.6 (0 to 31.17) | 8.09 (7.41 to 8.66) | 21.53 (0 to 50.59) |
| 63 | 1.89 (1.39 to 2.5) | 3.42 (0 to 13.63) | 2.97 (2.46 to 3.42) | 10.7 (0 to 34.11) | 9.03 (8.28 to 9.67) | 23.78 (0 to 54.67) |
| 64 | 2.09 (1.54 to 2.77) | 3.78 (0 to 14.93) | 3.29 (2.72 to 3.78) | 11.77 (0 to 36.84) | 9.95 (9.12 to 10.65) | 25.91 (0 to 58.33) |
| 65 | 2.29 (1.68 to 3.03) | 4.13 (0 to 16.18) | 3.59 (2.98 to 4.13) | 12.8 (0 to 39.37) | 10.83 (9.94 to 11.59) | 27.92 (0 to 61.61) |
| 66 | 2.56 (1.89 to 3.38) | 4.61 (0 to 17.87) | 4.02 (3.33 to 4.61) | 14.21 (0 to 42.69) | 12.04 (11.06 to 12.89) | 30.61 (0 to 65.76) |
| 67 | 2.82 (2.09 to 3.73) | 5.08 (0 to 19.47) | 4.43 (3.68 to 5.08) | 15.56 (0 to 45.69) | 13.2 (12.13 to 14.13) | 33.09 (0 to 69.32) |
| 68 | 3.08 (2.28 to 4.07) | 5.54 (0 to 20.98) | 4.83 (4.02 to 5.54) | 16.84 (0 to 48.4) | 14.31 (13.15 to 15.31) | 35.38 (0 to 72.38) |
| 69 | 3.33 (2.47 to 4.39) | 5.98 (0 to 22.4) | 5.22 (4.34 to 5.98) | 18.06 (0 to 50.86) | 15.37 (14.14 to 16.45) | 37.5 (0 to 75.03) |
| 70 | 3.57 (2.65 to 4.71) | 6.41 (0 to 23.75) | 5.59 (4.66 to 6.4) | 19.23 (0 to 53.09) | 16.39 (15.08 to 17.54) | 39.46 (0 to 77.31) |
| 71 | 3.78 (2.81 to 4.99) | 6.78 (0 to 24.88) | 5.92 (4.94 to 6.77) | 20.22 (0 to 54.89) | 17.26 (15.89 to 18.47) | 41.06 (0 to 79.07) |
| 72 | 3.99 (2.97 to 5.25) | 7.13 (0 to 25.95) | 6.23 (5.21 to 7.13) | 21.17 (0 to 56.54) | 18.09 (16.66 to 19.36) | 42.54 (0 to 80.6) |
| 73 | 4.18 (3.12 to 5.51) | 7.47 (0 to 26.95) | 6.53 (5.46 to 7.47) | 22.06 (0 to 58.03) | 18.87 (17.39 to 20.2) | 43.9 (0.79 to 81.93) |
| 74 | 4.37 (3.26 to 5.75) | 7.8 (0 to 27.88) | 6.82 (5.71 to 7.79) | 22.9 (0 to 59.38) | 19.62 (18.08 to 20.99) | 45.15 (1.59 to 83.08) |
| 75 | 4.56 (3.4 to 5.99) | 8.12 (0 to 28.77) | 7.1 (5.95 to 8.11) | 23.7 (0 to 60.61) | 20.33 (18.74 to 21.75) | 46.3 (2.37 to 84.08) |
| 76 | 4.7 (3.52 to 6.17) | 8.37 (0 to 29.45) | 7.32 (6.14 to 8.36) | 24.32 (0 to 61.54) | 20.89 (19.26 to 22.35) | 47.17 (3 to 84.81) |
| 77 | 4.84 (3.62 to 6.35) | 8.61 (0 to 30.09) | 7.53 (6.32 to 8.59) | 24.91 (0 to 62.38) | 21.41 (19.74 to 22.91) | 47.96 (3.6 to 85.44) |
| 78 | 4.97 (3.72 to 6.52) | 8.83 (0 to 30.68) | 7.73 (6.49 to 8.82) | 25.46 (0 to 63.14) | 21.9 (20.2 to 23.43) | 48.69 (4.17 to 85.98) |
| 79 | 5.09 (3.82 to 6.68) | 9.04 (0 to 31.23) | 7.92 (6.66 to 9.03) | 25.97 (0 to 63.83) | 22.36 (20.63 to 23.92) | 49.35 (4.7 to 86.46) |
| 80 | 5.21 (3.91 to 6.83) | 9.25 (0 to 31.74) | 8.09 (6.81 to 9.23) | 26.44 (0 to 64.46) | 22.79 (21.03 to 24.38) | 49.95 (5.21 to 86.88) |
| 81 | 5.29 (3.97 to 6.93) | 9.38 (0 to 32.07) | 8.21 (6.91 to 9.36) | 26.75 (0 to 64.85) | 23.07 (21.29 to 24.68) | 50.32 (5.54 to 87.12) |
| 82 | 5.36 (4.03 to 7.02) | 9.5 (0 to 32.37) | 8.32 (7.01 to 9.48) | 27.03 (0 to 65.2) | 23.32 (21.52 to 24.95) | 50.66 (5.85 to 87.34) |
| 83 | 5.43 (4.08 to 7.1) | 9.61 (0 to 32.64) | 8.42 (7.09 to 9.59) | 27.29 (0 to 65.51) | 23.56 (21.74 to 25.2) | 50.97 (6.13 to 87.53) |
| 84 | 5.49 (4.13 to 7.18) | 9.72 (0 to 32.89) | 8.51 (7.17 to 9.69) | 27.53 (0 to 65.79) | 23.77 (21.94 to 25.43) | 51.24 (6.39 to 87.69) |
| 85 | 5.54 (4.17 to 7.25) | 9.81 (0 to 33.12) | 8.6 (7.25 to 9.79) | 27.75 (0 to 66.04) | 23.97 (22.13 to 25.64) | 51.49 (6.63 to 87.84) |
