# Supplementary material for: Polygenic Risk Score Modifies Prostate Cancer Risk of Pathogenic Variants in Men of African Ancestry
Source: Cancer Res Commun. 2023 Dec 14;3(12):2544–50. doi: 10.1158/2767-9764.CRC-23-0022 (PMC10720390; doi:10.1158/2767-9764.CRC-23-0022)
Supplement: Supplementary Table 21 — Absolute risk of metastatic PCa by PRS and P/LP/D variants in BRCA2, ATM, NBN, and PALB2 combined in African ancestry men. [file crc-23-0022-s22.docx]

**Supplementary Table 21.** Absolute risk of metastatic PCa by PRS and P/LP/D variants in *BRCA2*, *ATM*, *NBN*, and *PALB2* combined in African ancestry men.

| **Absolute Risk(%) and 95% CI by Combined PRS and Carrier Status Categories** | | | | | | |
| --- | --- | --- | --- | --- | --- | --- |
| **Age** | **Low PRS Non−Carrier** | **Low PRS Carrier** | **Intermediate PRS Non−Carrier** | **Intermediate PRS Carrier** | **High PRS Non−Carrier** | **High PRS Carrier** |
| 40 | 0 (0 to 0) | 0 (0 to 0) | 0 (0 to 0) | 0 (0 to 0) | 0 (0 to 0) | 0 (0 to 0) |
| 41 | 0 (0 to 0) | 0 (0 to 0) | 0 (0 to 0) | 0 (0 to 0) | 0 (0 to 0) | 0 (0 to 0) |
| 42 | 0 (0 to 0) | 0 (0 to 0) | 0 (0 to 0) | 0 (0 to 0) | 0 (0 to 0) | 0 (0 to 0) |
| 43 | 0 (0 to 0) | 0 (0 to 0) | 0 (0 to 0) | 0 (0 to 0) | 0 (0 to 0) | 0 (0 to 0) |
| 44 | 0 (0 to 0) | 0 (0 to 0) | 0 (0 to 0) | 0 (0 to 0) | 0 (0 to 0) | 0 (0 to 0) |
| 45 | 0 (0 to 0) | 0 (0 to 0) | 0 (0 to 0) | 0 (0 to 0) | 0 (0 to 0) | 0 (0 to 0) |
| 46 | 0 (0 to 0) | 0.01 (0 to 0.06) | 0 (0 to 0) | 0.03 (0 to 0.12) | 0.01 (0.01 to 0.01) | 0.07 (0 to 0.3) |
| 47 | 0 (0 to 0) | 0.01 (0 to 0.11) | 0 (0 to 0.01) | 0.05 (0 to 0.23) | 0.01 (0.01 to 0.02) | 0.13 (0 to 0.6) |
| 48 | 0 (0 to 0.01) | 0.02 (0 to 0.16) | 0.01 (0 to 0.01) | 0.08 (0 to 0.35) | 0.02 (0.02 to 0.03) | 0.19 (0 to 0.89) |
| 49 | 0 (0 to 0.01) | 0.02 (0 to 0.22) | 0.01 (0.01 to 0.01) | 0.1 (0 to 0.47) | 0.03 (0.02 to 0.03) | 0.26 (0 to 1.18) |
| 50 | 0.01 (0 to 0.01) | 0.03 (0 to 0.27) | 0.01 (0.01 to 0.01) | 0.13 (0 to 0.58) | 0.04 (0.03 to 0.04) | 0.32 (0 to 1.47) |
| 51 | 0.01 (0 to 0.01) | 0.05 (0 to 0.42) | 0.02 (0.01 to 0.02) | 0.2 (0 to 0.89) | 0.06 (0.04 to 0.07) | 0.5 (0 to 2.27) |
| 52 | 0.01 (0.01 to 0.02) | 0.06 (0 to 0.56) | 0.02 (0.01 to 0.03) | 0.27 (0 to 1.2) | 0.07 (0.05 to 0.09) | 0.67 (0 to 3.05) |
| 53 | 0.02 (0.01 to 0.02) | 0.08 (0 to 0.71) | 0.03 (0.02 to 0.04) | 0.33 (0 to 1.51) | 0.09 (0.07 to 0.11) | 0.84 (0 to 3.81) |
| 54 | 0.02 (0.01 to 0.03) | 0.1 (0 to 0.85) | 0.04 (0.02 to 0.04) | 0.4 (0 to 1.81) | 0.11 (0.08 to 0.13) | 1.01 (0 to 4.57) |
| 55 | 0.02 (0.01 to 0.03) | 0.11 (0 to 0.99) | 0.04 (0.03 to 0.05) | 0.47 (0 to 2.11) | 0.13 (0.09 to 0.16) | 1.18 (0 to 5.32) |
| 56 | 0.03 (0.01 to 0.04) | 0.15 (0 to 1.3) | 0.05 (0.03 to 0.07) | 0.61 (0 to 2.75) | 0.17 (0.12 to 0.2) | 1.54 (0 to 6.91) |
| 57 | 0.04 (0.02 to 0.05) | 0.18 (0 to 1.59) | 0.07 (0.04 to 0.08) | 0.75 (0 to 3.38) | 0.21 (0.15 to 0.25) | 1.89 (0 to 8.45) |
| 58 | 0.04 (0.02 to 0.06) | 0.21 (0 to 1.88) | 0.08 (0.05 to 0.1) | 0.89 (0 to 4) | 0.25 (0.18 to 0.3) | 2.24 (0 to 9.95) |
| 59 | 0.05 (0.02 to 0.07) | 0.25 (0 to 2.17) | 0.09 (0.06 to 0.11) | 1.03 (0 to 4.6) | 0.29 (0.21 to 0.34) | 2.58 (0 to 11.41) |
| 60 | 0.06 (0.02 to 0.08) | 0.28 (0 to 2.45) | 0.1 (0.06 to 0.13) | 1.16 (0 to 5.2) | 0.33 (0.24 to 0.39) | 2.92 (0 to 12.83) |
| 61 | 0.07 (0.03 to 0.1) | 0.34 (0 to 3.02) | 0.13 (0.08 to 0.16) | 1.44 (0 to 6.39) | 0.41 (0.29 to 0.48) | 3.59 (0 to 15.66) |
| 62 | 0.08 (0.03 to 0.12) | 0.41 (0 to 3.57) | 0.15 (0.09 to 0.19) | 1.71 (0 to 7.53) | 0.48 (0.35 to 0.57) | 4.25 (0 to 18.33) |
| 63 | 0.09 (0.04 to 0.14) | 0.47 (0 to 4.1) | 0.17 (0.11 to 0.22) | 1.97 (0 to 8.64) | 0.56 (0.41 to 0.66) | 4.89 (0 to 20.86) |
| 64 | 0.11 (0.05 to 0.16) | 0.53 (0 to 4.62) | 0.2 (0.12 to 0.25) | 2.22 (0 to 9.72) | 0.63 (0.46 to 0.74) | 5.51 (0 to 23.25) |
| 65 | 0.12 (0.05 to 0.18) | 0.59 (0 to 5.13) | 0.22 (0.14 to 0.28) | 2.47 (0 to 10.75) | 0.7 (0.52 to 0.83) | 6.12 (0 to 25.52) |
| 66 | 0.13 (0.06 to 0.2) | 0.67 (0 to 5.73) | 0.25 (0.16 to 0.31) | 2.77 (0 to 12) | 0.79 (0.58 to 0.93) | 6.85 (0 to 28.2) |
| 67 | 0.15 (0.06 to 0.22) | 0.74 (0 to 6.32) | 0.27 (0.17 to 0.35) | 3.07 (0 to 13.19) | 0.87 (0.65 to 1.03) | 7.55 (0 to 30.68) |
| 68 | 0.16 (0.07 to 0.24) | 0.81 (0 to 6.88) | 0.3 (0.19 to 0.38) | 3.35 (0 to 14.32) | 0.95 (0.71 to 1.12) | 8.23 (0 to 32.99) |
| 69 | 0.17 (0.08 to 0.26) | 0.88 (0 to 7.42) | 0.32 (0.21 to 0.41) | 3.63 (0 to 15.4) | 1.03 (0.77 to 1.21) | 8.88 (0 to 35.14) |
| 70 | 0.19 (0.08 to 0.28) | 0.94 (0 to 7.93) | 0.35 (0.22 to 0.44) | 3.89 (0 to 16.44) | 1.11 (0.83 to 1.3) | 9.5 (0 to 37.13) |
| 71 | 0.2 (0.09 to 0.3) | 1.02 (0 to 8.57) | 0.38 (0.24 to 0.48) | 4.22 (0 to 17.7) | 1.21 (0.91 to 1.41) | 10.27 (0 to 39.49) |
| 72 | 0.22 (0.1 to 0.33) | 1.1 (0 to 9.17) | 0.41 (0.26 to 0.51) | 4.54 (0 to 18.89) | 1.3 (0.98 to 1.52) | 11 (0 to 41.63) |
| 73 | 0.23 (0.1 to 0.35) | 1.18 (0 to 9.74) | 0.43 (0.28 to 0.55) | 4.84 (0 to 20) | 1.39 (1.06 to 1.62) | 11.7 (0 to 43.57) |
| 74 | 0.25 (0.11 to 0.37) | 1.25 (0 to 10.28) | 0.46 (0.3 to 0.58) | 5.13 (0 to 21.05) | 1.47 (1.12 to 1.72) | 12.36 (0 to 45.32) |
| 75 | 0.26 (0.12 to 0.39) | 1.32 (0 to 10.79) | 0.49 (0.32 to 0.62) | 5.41 (0 to 22.04) | 1.56 (1.19 to 1.81) | 12.99 (0 to 46.91) |
| 76 | 0.28 (0.12 to 0.42) | 1.4 (0 to 11.35) | 0.51 (0.34 to 0.65) | 5.71 (0 to 23.1) | 1.65 (1.27 to 1.91) | 13.66 (0 to 48.55) |
| 77 | 0.29 (0.13 to 0.44) | 1.47 (0 to 11.86) | 0.54 (0.36 to 0.69) | 6 (0 to 24.07) | 1.73 (1.34 to 2.01) | 14.3 (0 to 50) |
| 78 | 0.31 (0.14 to 0.46) | 1.54 (0 to 12.34) | 0.57 (0.38 to 0.72) | 6.27 (0 to 24.97) | 1.81 (1.41 to 2.1) | 14.89 (0 to 51.29) |
| 79 | 0.32 (0.14 to 0.48) | 1.6 (0 to 12.79) | 0.59 (0.39 to 0.75) | 6.52 (0 to 25.8) | 1.89 (1.47 to 2.19) | 15.44 (0 to 52.42) |
| 80 | 0.33 (0.15 to 0.5) | 1.67 (0 to 13.21) | 0.61 (0.41 to 0.78) | 6.76 (0 to 26.56) | 1.96 (1.53 to 2.27) | 15.96 (0 to 53.43) |
| 81 | 0.35 (0.15 to 0.52) | 1.73 (0 to 13.63) | 0.64 (0.43 to 0.81) | 7.01 (0 to 27.34) | 2.04 (1.6 to 2.36) | 16.48 (0 to 54.41) |
| 82 | 0.36 (0.16 to 0.54) | 1.79 (0 to 14.02) | 0.66 (0.44 to 0.84) | 7.24 (0 to 28.03) | 2.11 (1.66 to 2.44) | 16.96 (0 to 55.25) |
| 83 | 0.37 (0.16 to 0.56) | 1.85 (0 to 14.37) | 0.68 (0.46 to 0.86) | 7.45 (0 to 28.65) | 2.17 (1.71 to 2.51) | 17.4 (0 to 55.97) |
| 84 | 0.38 (0.17 to 0.57) | 1.9 (0 to 14.69) | 0.7 (0.47 to 0.89) | 7.64 (0 to 29.21) | 2.23 (1.76 to 2.58) | 17.8 (0 to 56.6) |
| 85 | 0.39 (0.17 to 0.59) | 1.94 (0 to 14.97) | 0.72 (0.48 to 0.91) | 7.82 (0 to 29.71) | 2.29 (1.81 to 2.64) | 18.16 (0 to 57.13) |
